# Navigating the glycomics landscape with CE-MS: advances in sample preparation and analytical strategies

**DOI:** 10.1039/d6an00304d

**Published:** 2026-06-30

**Authors:** Karthika Korumadathil Shaji, Peter L. Horvatovich, Guinevere S. M. Lageveen-Kammeijer

**Affiliations:** a University of Groningen, Groningen Research Institute of Pharmacy, Analytical Biochemistry 9700 AD Groningen the Netherlands g.s.m.kammeijer@rug.nl

## Abstract

Glycosylation is one of the most structurally diverse and biologically consequential co- and post-translational modifications, yet its analytical characterisation remains challenging due to extensive isomerism, microheterogeneity, branching structure and the presence of labile residues. Among the available analytical platforms, capillary electrophoresis (CE), particularly when coupled to mass spectrometry (CE-MS), offers exceptional separation efficiency at nanolitre sample loadings and can resolve glycan variants that remain obscured in conventional LC- or MALDI-based workflows. This review provides a comprehensive overview of recent advances that have expanded the utility of CE and CE-MS in glycomics. We discuss practical considerations in enzymatic and chemical glycan release and highlight how the workflow format and clean-up influence recovery, quantitative precision and downstream compatibility. A major section is dedicated to the critical evaluation of major reducing-end derivatisation chemistries, including reductive amination, hydrazide and Michael-addition labelling, stable isotope, isobaric, and emerging instant-labelling strategies as well as permethylation, focusing on how labelling modulates electrophoretic mobility, isomer resolution, ionisation efficiency and MS/MS fragmentation. We outline current CE-MS methodologies, focusing on background electrolyte design, capillary coatings, sample injection modes, and the latest developments in sheath-flow, sheathless, nanoflow, and microfluidic interfaces. Performance benchmarks, including sensitivity, isomer resolution, robustness, and quantitative precision, are evaluated alongside recent innovations such as dopant enriched gases and integrated CE-MS cartridges. Finally, we assess the opportunities and remaining barriers for the broader adoption of CE-MS in biomedical, clinical, and biopharmaceutical glycomics. Continued advances in MS interface design, automation, and MS-compatible labelling chemistries are expected to further transform CE-MS into a routinely and widely deployable platform for high-resolution glycan characterisation.

## Abstract 2

CE-MS has emerged as a powerful platform for glycomics. Advances in sample preparation, derivatization chemistry, and MS interfacing enable high-resolution glycan separation and comprehensive structural characterization from composition to topology.

## Graphical abstract

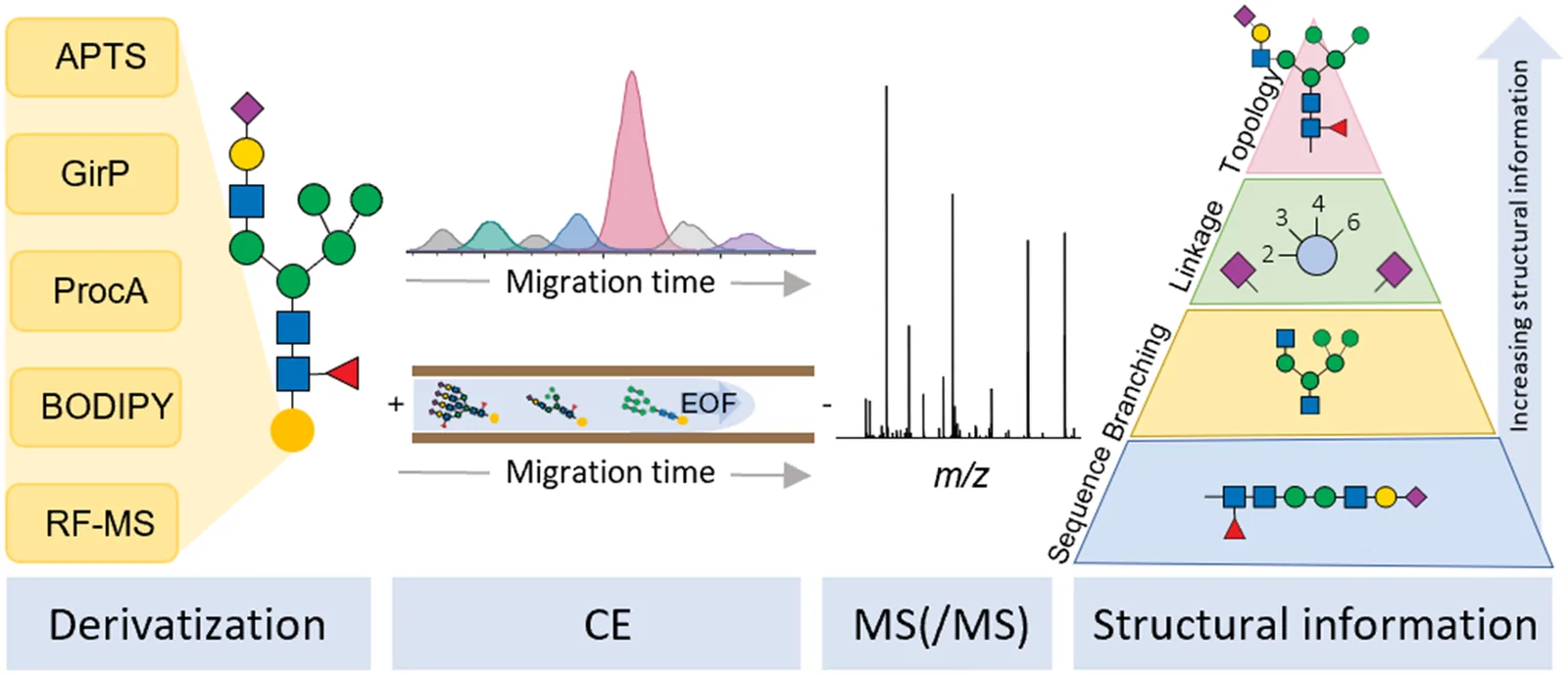

## 1. Introduction

**Fig. 1:**
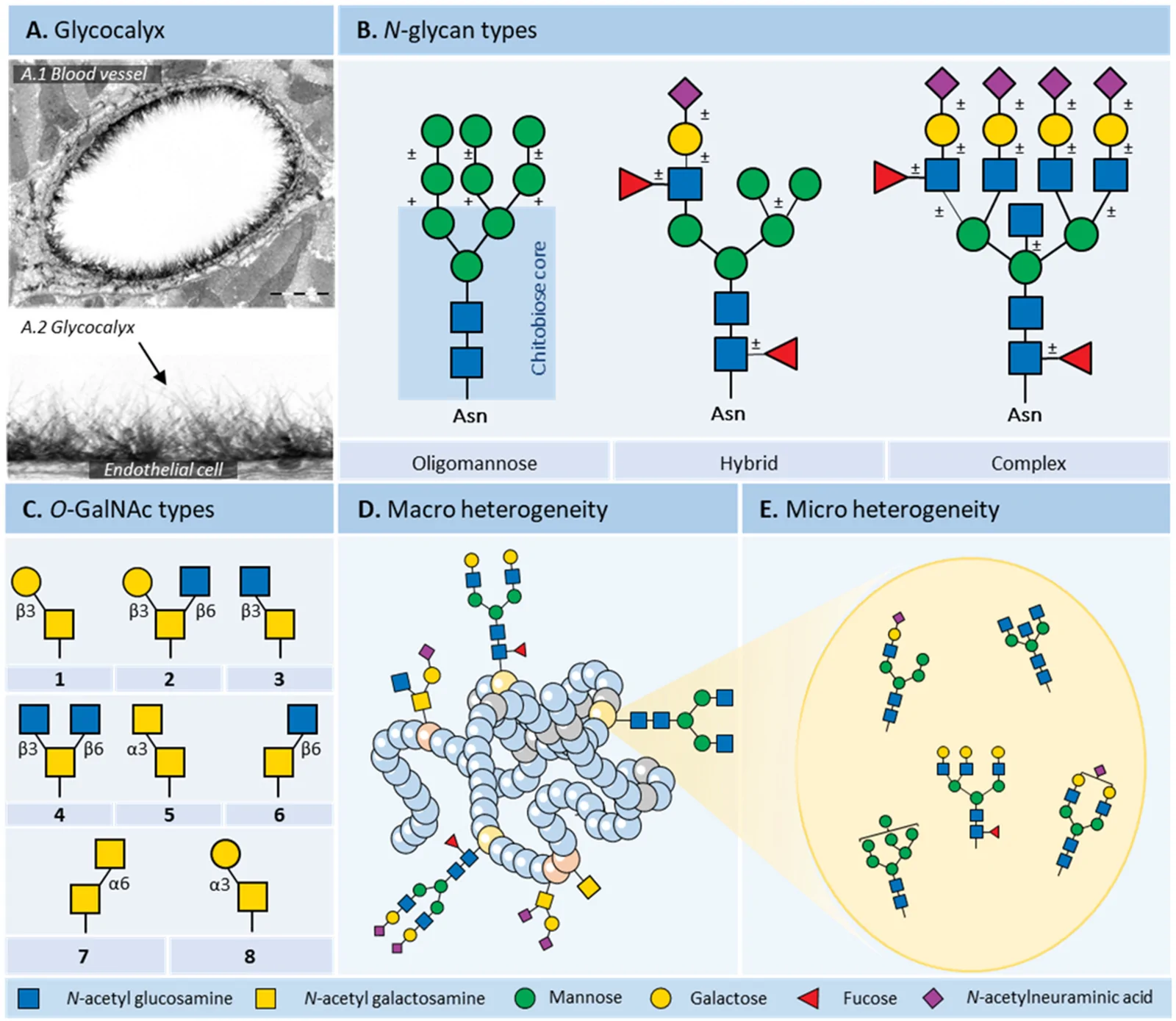
Overview of glycan biology and structural heterogeneity relevant to glycoanalytics. (A) The endothelial glycocalyx visualized by electron microscopy (A.1; Alcian blue 8GX staining) with a magnified view (A.2) highlighting the cell surface-associated carbohydrate layer. (B) Major classes of *N*-glycans (oligomannose, hybrid and complex) sharing the conserved chitobiose core (GlcNAc_2_Man_3_) linked to Asn. (C) Core structures of mucin-type *O*-glycans (*O*-GalNAc; cores 1–8). (D) Macroheterogeneity: variation in site occupancy and/or complete glycoform distributions across proteins or biological contexts. (E) Microheterogeneity: site-specific variability in glycoform composition at an occupied glycosylation site. Fig. 1A is reproduced from van den Berg, Vink and Spaan with permission from Wolters Kluwer Health, Inc.^6^

Glycosylation is one of the most complex and diverse co- and post-translational modifications of proteins and occurs in the endoplasmic reticulum (ER) as well as in the Golgi apparatus.^1,2^ It plays a crucial role in protein stability, folding, trafficking and cellular recognition.^3^ The glycocalyx (Fig. 1A), a carbohydrate-rich, multifunctional layer on the cell surface, is mainly composed of glycoproteins, proteoglycans, and glycolipids, and serves as a central mediator of cell membrane protection as well as cell–cell and cell–matrix interactions. Subtle changes in glycan structure, such as the transition from α2-3 to α2-6 sialylation, have been directly linked to disease onset, progression, and therapy resistance.^4^ Consequently, glycosylation patterns are increasingly recognized as valuable biomarkers for diagnostics, prognostics, and therapeutic monitoring.^5^

These disease-relevant alterations arise from the inherent complexity of glycan biosynthesis, which is a dynamic molecular process jointly influenced by genetic factors, the internal state of the cell and organism, and environmental conditions.^7^ Unlike proteins or nucleic acids, glycans (complex carbohydrates) are not template-driven but are instead built by the coordinated action of ∼200 glycosyltransferases, which assemble and remodel glycans from a limited set of monosaccharides. In humans, the most common building blocks include hexoses (mannose [Man], glucose [Glc], galactose [Gal]), fucose [Fuc], *N*-acetylhexosamines (*N*-acetylglucosamine [GlcNAc], *N*-acetylgalactosamine [GalNAc]), and sialic acids (*N*-acetyl neuraminic acid [NeuAc]) for protein glycosylation. These glycans are glycosidically linked in either linear or branched configurations, as well as with α- or β-linkages, and have defined hydroxyl group positions,^2^ forming structures ranging from a single monosaccharide to highly complex glycans with more than 20 residues (Fig. 1). Even subtle modifications in branching, linkage, or residue composition can alter protein function, with direct implications in aging, cancer, diabetes, Alzheimer's disease, and cardiovascular diseases.^8^

Protein glycosylation is broadly divided into two major classes: N-linked and O-linked. In N-linked glycosylation, a conserved precursor (Glc_3_Man_9_GlcNAc_2_) is transferred from a dolichol phosphate carrier to the side-chain nitrogen atom of an asparagine residue within the consensus sequence (Asn-Xxx-Ser/Thr, where Xxx can be any amino acid except proline).^9^ This process occurs either co- or post-translationally (*i.e.* during the protein translation and folding process or afterwards) in the ER, where the product further undergoes initial trimming and remodeling before further maturation of the glycan structure in the Golgi apparatus (post-translational).^1^*N*-Glycans share a defined chitobiose core composed of five monosaccharides (GlcNAc_2_Man_3_), and extension of this core yields the oligomannose, complex, or hybrid subclasses (Fig. 1B). O-Linked glycosylation, in contrast, occurs exclusively post-translationally in the Golgi apparatus and involves the attachment of a sugar moiety to the oxygen atom of a serine or threonine residue, without a strict requirement for a consensus sequence on the protein amino acid sequence backbone. The most prevalent *O*-glycan type is mucin-type *O*-glycans (*O*-GalNAc), which can be classified into eight core structures (Fig. 1C) and can be extended into numerous elongated and branched structures.^10^

Beyond these biosynthetic rules, glycans exhibit two additional layers of variation: macroheterogeneity^11,12^ (Fig. 1D), describing differences in glycoforms across proteins, and microheterogeneity^11,12^ (Fig. 1C), reflecting site-specific variability within a protein sequence. Together, this biosynthetic and structural diversity underpins the functional roles of glycans while illustrating their extraordinary complexity (Fig. 1), which sets a highly challenging goal for analytical platforms. Macro- and microheterogeneity together contribute to the remarkable diversity of glycan structures observed across cells and tissues. At the macroheterogeneity level, site occupancy varies because the efficiency of glycan attachment to specific sequences depends on the local cellular glycosylation machinery and enzyme composition, activity, and spatial organization, as well as the 3D structure of the target protein such that a site may be fully glycosylated in one context but only partially or completely unoccupied in another. At the microheterogeneity level, even when a site is occupied, the specific glycoforms generated are shaped by dynamic factors including the repertoire and relative activities of glycosyltransferases and glycosidases, the availability of nucleotide–sugar donors, and Golgi compartmentalization. Because these biochemical and regulatory conditions fluctuate with cell type, developmental stage, metabolic state, and environmental or pathological cues, both site occupancy and the structural composition of glycans at occupied sites are continuously remodeled, producing context-dependent glycosylation patterns that modulate protein function in a highly dynamic manner.^13–15^ The systematic study of these structures and their functions is termed glycomics, a field that aims to comprehensively characterize the glycan repertoire of cells, tissues, and organisms. In this review, we address glycomics through the lens of capillary electrophoresis (CE)-based glycoanalytics (released-glycan profiling), rather than a comprehensive overview of glycoproteomics and other glycosylation analysis strategies. The enormous structural diversity and the presence of isomeric forms make glycomics far more analytically demanding than proteomics or genomics. Sensitive, high-resolution workflows that can separate closely related isomers while providing quantitative and structural readouts are therefore essential.

Among separation-based platforms, capillary electrophoresis (CE) has attracted attention as a powerful approach for glycoanalytics.^16,17^ With intrinsically high separation efficiency, nanoliter-scale sample consumption, and operation in aqueous buffers, CE is well suited to resolving isomeric glycans and to analyzing highly polar, weakly retained species that can be challenging in conventional RP-LC workflows, while also helping to preserve labile glycans (*e.g.*, sialylated species). When coupled to mass spectrometry (CE-MS), CE enables sensitive identification and structure elucidation,^18,19^ while CE with laser-induced fluorescence (CE-LIF) remains highly valuable as a robust, quantitative glycan profiling analytical platform.^20,21^

A critical factor that underpins the success of many CE-based approaches is glycan labelling. Since native glycans lack strong chromophores and often ionize poorly, chemical and enzymatic derivatization strategies are widely employed to enhance sensitivity, CE separation resolution, and quantitation.^22,23^ Labelling chemistries also influence fragmentation behavior, stability, sensitivity and isomeric separation, and their impact can vary across detection types. Nevertheless, label-free CE approaches, particularly CE-MS in negative ion mode, have also proved to be powerful for analyzing underivatized glycans, avoiding additional sample preparation steps and preserving native structures.^24^ Recent advances in MS interfaces, labelling chemistry, and microfluidic integration are enhancing the sensitivity, robustness and practicality of CE-based workflows, making it a powerful separation tool to study glycosylation in complex clinical samples.

In this review, we focus on glycan labelling strategies and their application in CE-based workflows, while also addressing several key aspects of label-free approaches as a complementary tool. We critically assess existing derivatization chemistries, their impact on sensitivity and structural elucidation in CE-LIF and CE-MS, and how they compare to other analytical platforms such as LC-MS and matrix assisted laser desorption ionization (MALDI)-MS. We also highlight emerging chemistries and provide a forward-looking perspective on the unsolved challenges for CE analysis, particularly CE-MS, to transition from a specialized technique to a more broadly applied platform in the glycoscience field.

## 2. Analytical challenges in glycomics

The analysis of glycans is uniquely demanding because monosaccharide composition does not uniquely define chemical structure and biologically relevant differences often arise from subtle linkage, branching and positional changes. As a result, isomeric glycans can share identical monosaccharide compositions and mass-to-charge (*m*/*z*) values and remain indistinguishable by MS alone without prior separation or diagnostic fragmentation. In addition, different monosaccharide combinations can be isobaric, such that distinct compositions yield identical nominal (and in some cases near-identical exact) masses. Labile substituents such as sialic acids and sulfates may undergo partial loss during sample preparation, ionization, or fragmentation, further confounding identification and quantitation. Because many native glycans ionize inefficiently, their detection is also more susceptible to matrix-driven ion suppression, particularly in complex biological samples. At the protein level, macro- and microheterogeneity further expand glycosylation diversity, so that even small sample sets can contain hundreds of glycoforms requiring discrimination at high sensitivity.

Beyond structural complexity, quantitative reproducibility remains a major challenge. Glycan abundances can differ between biological states, yet variability introduced by sample preparation, instrument parameters, and inter-laboratory workflows can obscure these changes. Batch effects and the lack of widely accepted reference standards hinder comparability across studies, and remain barriers to broad harmonization and regulatory-grade deployment, even though glycosylation is already clinically impactful (*e.g.* congenital disorders of glycosylation (CDG) diagnostics and glycoform-resolved assays such as AFP-L3 ^25–27^) and many routine immunoassays rely on glycan-dependent epitopes.^28–30^ Together, these challenges underscore the need for robust derivatization strategies and high-resolution analytical platforms that can deliver sensitive, reproducible, and quantitative glycan profiling.

## 3. Comparative overview of glycan analysis techniques

Detection is a key element of glycomics analytical approaches, with fluorescence- and MS-based approaches dominating current workflows (Table 1). Fluorescence detection (FLD or LIF) offers excellent sensitivity, reproducibility, and quantitative reliability, since fluorescent labelling produces a signal that is approximately proportional to glycan molar amount when labeling is near-quantitative and yields a consistent dye-to-glycan stoichiometry (typically ∼1 label per glycan). This enables quantification using external calibration with a single labeled reference compound (not necessarily a glycan), providing practical molar readouts across a wide range of glycans. However, fluorescence provides no direct structural information. In contrast, MS combines high sensitivity with the ability to distinguish analytes by their *m*/*z* ratio. Tandem MS adds fragmentation patterns that reveal linkages, branching, and substituents, enabling detailed structural characterization even at trace levels.^31–33^ The extent of obtainable information, however, depends strongly on how MS is coupled to ionization and separation analytical hardware.

**Table 1:** Comparative overview of major analytical techniques used in glycomics, including LC-, IM-MS-, MALDI-, NMR-, and CE-based approaches

Techniques		Sensitivity	Reproducibility	Quantitative performance	Structural information	Isomer resolution	Advantages	Limitations	Ref.
LC	LC-FLD	++	+++	+++	−	+	Robust quantitation	No structural information	22 and 34–40
							High reproducibility	Labeling required	
	LC-MS (RP-LC)	+++	++	++	+++	+	High sensitivity	Hydrophobic labeling required	41, 42, 39 and 43
							Detailed structural characterization	Limited retention of native glycans	
	LC-MS (HILIC)	+++	+++	+++	+++	++	Excellent glycan retention	Moderate isomer resolution	22, 41, 39, 40 and 44
							Robust quantitative analysis	Longer analysis times	
	LC-MS (PGC)	+++	+	++	+++	+++	Superior isomer separation	Lower reproducibility	45, 39, 40, 46 and 47
							Native glycan analysis	Method-dependent retention	
IM-MS		++	++	++	+++	++	CCS-based identification	Limited CCS databases	31, 34 and 48–52
							Enhanced isomer discrimination	Complex data analysis	
MALDI	MALDI-TOF-MS	+	+	++	+	−	Rapid high-throughput analysis	Limited isomer resolution	53, 36 and 54–57
							Simple spectral interpretation	Semi-quantitative performance	
	MALDI-MSI	+	+	−	−	−	Spatial glycan mapping	Limited quantitation	58 and 59–63
							Tissue-specific information	Limited isomer separation	
NMR		−	+++	++	+++	+++	Comprehensive structural elucidation	Low sensitivity	64, 65 and 66–69
							Non-destructive analysis	High sample requirement	
CE	CE-LIF	+++	+++	+++	−	+++	Robust quantitation	No structural information	34–36 and 70–72
							Excellent isomer resolution	Labeling required	
	CE-MS	+++	++	++	+++	+++	High sensitivity	Interface-dependent performance	73, 74, 75, 34, 76 and 77
							Detailed structural characterization	Method optimization required	

### 3.1 Liquid chromatography

LC is the most widely used separation technique in glycomics and is typically coupled to either fluorescence or mass spectrometric detection. With fluorescence detection (LC-FLD), glycans are derivatized to introduce a fluorescent tag, enabling robust, sensitive, and reproducible quantification.^78^ The proportionality of measured fluorescence signal and glycan molar concentration makes LC-FLD highly reliable and cost-effective for routine profiling. In contrast, LC-MS combines separation with the structural elucidation capabilities of MS described above. This makes LC-MS highly selective and capable of identifying glycans even at trace levels. Yet the LC-MS performance depends strongly on the stationary phase. RP-LC struggles to retain unmodified hydrophilic released glycans, necessitating hydrophobic labelling.^79^ Hydrophilic interaction chromatography (HILIC) improves retention of the glycans due to their polar properties and is widely applied in glycomics.^80^ Porous graphitized carbon (PGC) provides particularly strong retention and excellent isomer resolution and is therefore widely used for the analysis of underivatized (native) glycans. Retention on PGC arises from a combination of dispersive and polarizable surface interactions, which makes it effective at separating structurally similar species, including linkage and positional isomers that frequently co-elute on other phases. PGC is especially valuable for small and neutral glycans and for *O*-glycans, where isomer separation is often a primary analytical bottleneck.^45,81^ However, PGC separations can be sensitive to mobile-phase composition and column history, and strongly retained glycans may show longer run times or memory effects, which can impact robustness for high-throughput workflows.^45,82,83^ Overall, LC-FLD excels at quantitative profiling and LC-MS at structural depth.

### 3.2 Ion mobility mass spectrometry (IM-MS)

In recent years, ion mobility spectrometry (IM) combined with MS has emerged as a powerful tool for glycan analysis.^84^ Ion mobility spectrometry extends the resolving power of MS by separating ions in the gas phase according to their size and shape. Drift times can be converted into collision cross section (CCS) values, which provide reproducible descriptors of glycan conformation. CCS is a molecular property that, under controlled conditions, remains independent of instrument parameters and is directly related to an ion's shape. Because of this, CCS values can be stored in databases and used as an additional parameter to strengthen identification certainty, enabling more accurate and reliable structural characterization.^85^ This additional parameter strengthens isomer discrimination beyond *m*/*z* alone, offering complementary selectivity to LC and CE separations.^86^ IM-MS is a promising addition to the glycomics toolkit, though workflows and CCS libraries are not yet sufficiently mature for routine application.^85^ The rich conformational space of highly sulfated glycans such as proteoglycans can further complicate spectral interpretation, as a single structure may adopt multiple gas-phase conformers with distinct CCS values.

### 3.3 MALDI-TOF-MS

MALDI-TOF-MS is often described as a straightforward MS-based approach, with glycans co-crystallized with a matrix and ionized directly by laser irradiation. This simplicity enables high-throughput and rapid screening, while the predominance of singly charged ions simplifies spectra and access to higher mass ranges facilitates the analysis of larger glycans.^53,87^ In practice, however, the overall workflow is not always sample preparation free as derivatization is frequently required, particularly for sialic acids, to stabilize labile residues and prevent in-source fragmentation (see Section 5),^85^ adding additional preparation steps.^53^ Despite these caveats, MALDI remains attractive for screening applications where speed and simplicity are prioritized.

An important extension of MALDI is MALDI imaging (MSI), which applies the same principles directly to tissue sections. In this approach, thin tissue slices are prepared on conductive slides, and glycans (commonly *N*-glycans) are released enzymatically. Derivatization methods such as permethylation or reducing-end labelling may be introduced to improve sensitivity.^88^ A laser then ionizes the glycan matrix crystals in a raster pattern, generating mass spectra from a specific tissue slice location resulting as an image pixel, which are used to computationally reconstruct ion maps of glycan distributions in the original tissue. MALDI imaging has proved to be valuable for studying tissue-specific glycosylation patterns, particularly in cancer^89,90^ and neurodegenerative disease,^91,92^ and led to several successful biomarker discovery studies,^93–95^ highlighting key roles of glycans in complex diseases. This approach preserves spatial context while offering high molecular specificity. Nevertheless, challenges remain, including the difficulty in distinguishing isomeric glycans due to the lack of additional separation such as that provided by LC or CE and the relatively low ionization efficiency of many glycan species.

### 3.4 Nuclear magnetic resonance (NMR)

NMR is an effective technique that enables precise determination of anomeric configurations, linkage position, and substituents.^64^ Unlike MS- or fluorescence-based approaches, NMR can characterize glycans without derivations or labelling, and its non-destructive nature enables samples to be preserved for future analysis. These features make NMR uniquely valuable for detailed structural elucidation of relatively pure samples, which pose problems for analyzing the large number of glycans in complex biological samples. However, the method has low sensitivity and requires a high sample amount, samples with low molecular complexity, and long acquisition times.^96^ The generated complex data make interpretation challenging, which, combined with high operational costs and specific data interpretation expertise requirements, limit routine use of the method.^65^ As a result, NMR remains largely confined to specialized structural studies rather than large-scale glycomics profiling.^64,97^

### 3.5 Capillary electrophoresis (CE)

Within the current analytical landscape, CE distinguishes itself by offering a high separation efficiency with minimal sample requirements and streamlined sample preparation, typically limited to glycan release, optional labeling, and simple desalting/cleanup prior to analysis. With nanoliter (nL)-scale injections, CE is particularly effective at resolving closely related glycan isomers. As highlighted earlier on, fluorescence enables robust and quantitative profiling, providing highly reproducible molar quantification in the context of CE-LIF, with appropriate calibration, whereas CE-MS provides detailed structural information and isomer resolution, although with greater complexity and optimization demands. Innovations in interfaces to MS, such as sheathless electrospray designs, and microfluidic formats have substantially improved robustness and sensitivity.^73,98,99^ These advances have established CE as a strong complement to LC- and MALDI-based platforms and provide the foundation for a detailed discussion of CE principles, labelling strategies, and applications in the following sections.

## 4. Glycan release strategies

The release of glycans from glycoproteins is the critical first step in the glycomics workflow. Before enzymatic or chemical cleavage, proteins are typically subjected to denaturation, reduction and alkylation to increase the accessibility of glycans. Denaturants such as urea, guanidine hydrochloride or sodium dodecyl sulphate (SDS) unfold the tertiary structure, exposing covalent bonds for cleavage. Reducing agents such as dithiothreitol (DTT) and β-mercaptoethanol break disulphide bonds, while alkylating agents such as iodoacetamide or iodoacetic acid prevent their re-formation. These preparatory steps are essential for ensuring the efficient and reproducible release of glycans, particularly from folded or heavily cross-linked proteins.^17,22,100^ Two broad strategies are then employed for glycan release: enzymatic and chemical approaches. While both can in principle be applied to *N*- and *O*-glycans, enzymatic release is typically preferred for *N*-glycans, whereas chemical release remains essential for *O*-glycans.

### 4.1 Enzymatic release of *N*- and *O*-glycans

**Fig. 2:**
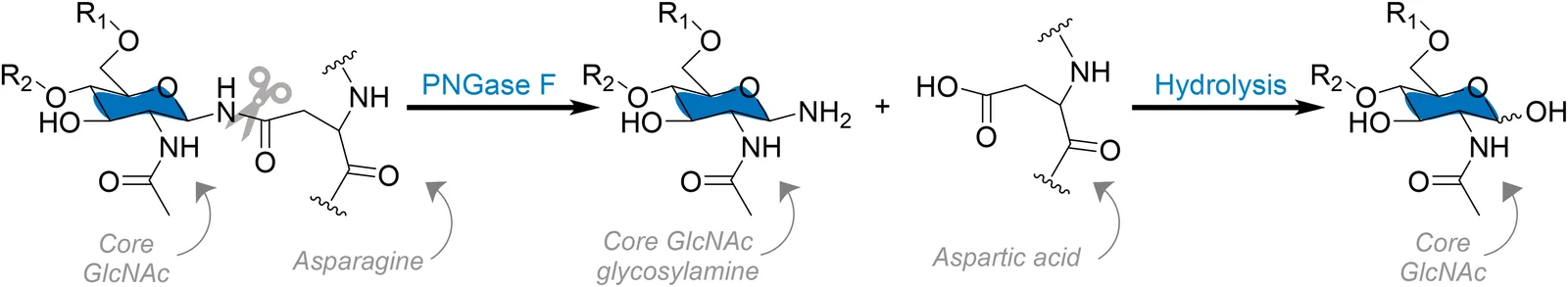
Enzymatic release of *N*-glycans by PNGase F. The enzyme PNGase F cleaves the bond between the innermost GlcNAc and the asparagine side chain, releasing the *N*-glycan and converting the glycosylated asparagine into aspartic acid (deamidated asparagine).

For *N*-glycans, PNGase F is the most commonly used endoglycosidase for mammalian samples, efficiently cleaving nearly all structures at the amide bond between the innermost GlcNAc and the asparagine side chain, thereby releasing the intact *N*-glycan and converting asparagine into aspartic acid *via* deamidation^12,101–103^ (Fig. 2). Its main limitation is the inability to act on glycans with core α1,3 fucosylation,^104^ a common feature in plants and insects. In such cases, PNGase A^104^ is required, which is an enzyme with broader specificity, but it operates more slowly and under acidic conditions, making workflows longer and sometimes less compatible with acid-sensitive proteins. Both enzymes provide highly specific release and generate intact reducing ends that are ideal for derivatization, and PNGase F preserves labile modifications such as sialylation. Limitations include steric hindrance from protein folding or extensive modification, particularly core fucosylation, dense glycosylation, or phosphorylation, which can reduce cleavage efficiency and can be prevented by denaturing the sample prior to enzymatic cleavage.^105,106^ More recently, engineered PNGase F variants, such as Rapid PNGase F,^107^ were introduced to accelerate release, improve compatibility with native conditions, and expand applicability to more challenging glycoproteins,^108^ including heavily glycosylated, hydrophobic, or structurally rigid proteins that are resistant to conventional denaturation-based workflows. These developments provide analysts with greater flexibility in balancing speed, specificity, and sample integrity.

For *O*-glycans, by contrast, enzymatic release remains far less developed. No broadly acting *O*-glycosidases exist, meaning that complete enzymatic release of *O*-glycans is not possible.^109,110^ Available enzymes are restricted to highly specific *O*-glycan cores or lead to truncated *O*-glycan species, (*e.g. O*-glycosidase can remove unsubstituted Galβ1-3GalNAc (“core 1”) structures, but cannot act on sialylated or extended *O*-glycans).^111,112^ Exoglycosidase arrays can be used to sequentially trim certain *O*-glycan epitopes, but these approaches are labor-intensive, provide an incomplete cleavage profile, and are better suited for structural confirmation than comprehensive release. As a result, enzymatic release plays only a minor role in *O*-glycan analysis, and chemical approaches (Section 4.2) remain the standard method for accessing the full repertoire of *O*-glycan structures. However, it is worth noting that recently, novel bacterial enzymes, termed POGases (peptide:*O*-glycosidases), with broader substrate specificity have been identified that are capable of releasing sialylated *O*-glycans from glycoproteins; this represents a promising step towards broader enzymatic *O*-glycan release, although it is not yet broadly available.^113^

### 4.2 Chemical release approaches

**Fig. 3:**
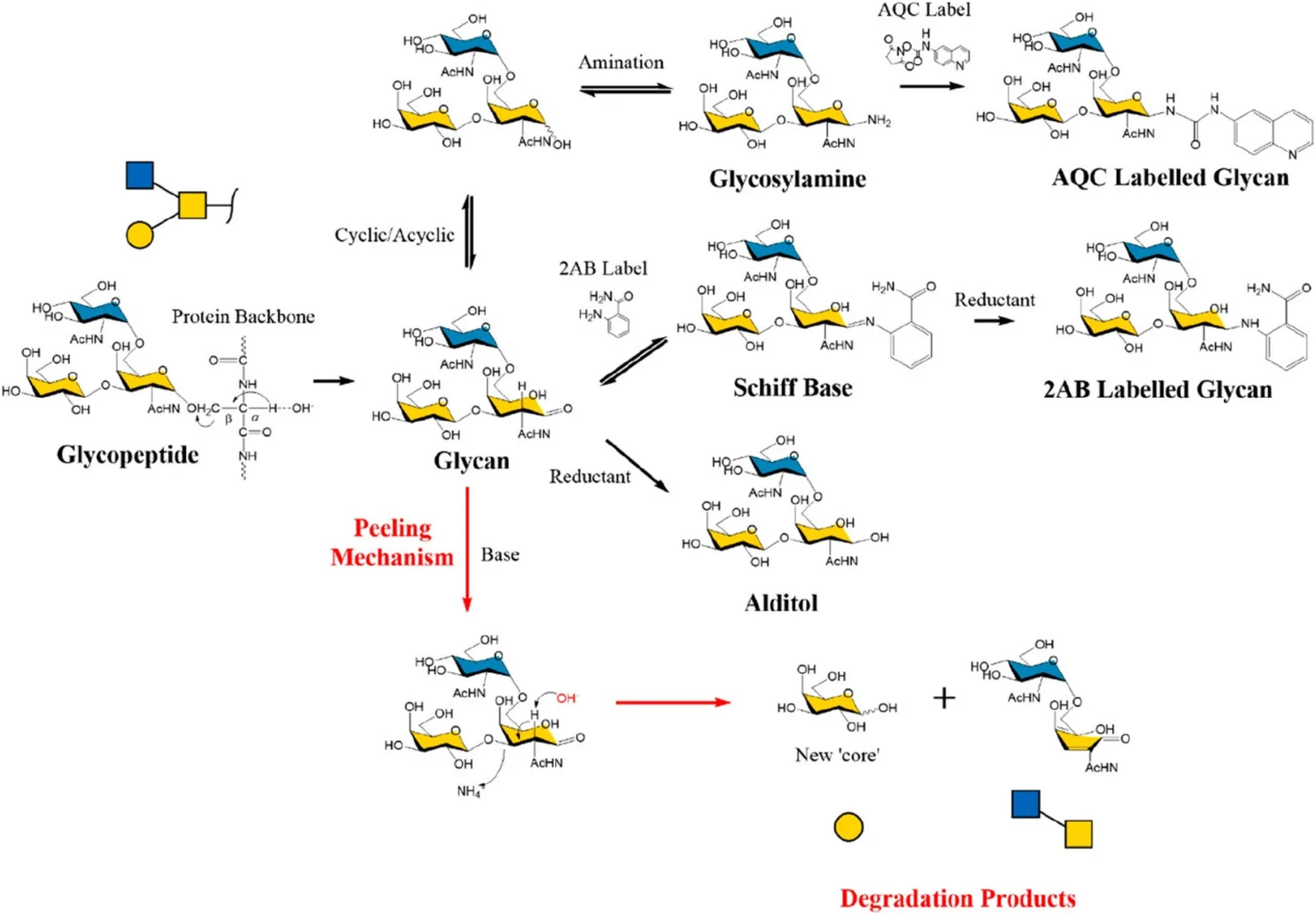
*O*-Glycan β-elimination, reducing-end chemistry, and peeling mechanism. Released glycans exist as cyclic/acyclic equilibrium forms; the acyclic form enables reductive amination (2-AB) or reduction to alditol, while the cyclic form enables glycosylamine-based labeling (AQC). Basic conditions promote peeling degradation, generating a new core and degradation products. Reproduced from Wilkinson and Saldova with permission from the American Chemical Society.^114^

Chemical methods are applicable to both *N*- and *O*-glycans, but their role differs.^111^ For *N*-glycans, chemical release was historically performed but these are now largely obsolete with the arrival of endoglycosidases such as PNGase F and A. These chemical methods often cause glycan modifications, incomplete release and recovery, and degradation of glycans (Fig. 3), making enzymatic release more suitable for *N*-glycan workflows.^110^

For *O*-glycans, in contrast, chemical release remains indispensable due to the lack of broadly acting endoglycosidase enzymes. Reductive β-elimination^112^ is the most widely used strategy, cleaving *O*-glycosidic bonds to release glycans. However, it carries the risk of “peeling” side reactions,^112^ which shorten glycans and therefore modify the original glycan composition of the analyzed sample and compromise reducing-end integrity, lowering the efficiency of subsequent labelling.^22,115,116^ Hydrazinolysis^112^ can also release *O*-glycans (and *N*-glycans), and it generally preserves reducing ends better than β-elimination. Yet, the harsh and hazardous conditions required increase the risk of damaging labile groups such as sialic acids and limit routine application. As with β-elimination, glycans released by hydrazinolysis are prone to modification. While reductive amination is the most common derivatization method following hydrazinolysis, alternative stabilization chemistries have been explored, including permethylation and pyrazolone-based Michael addition^22^ (see Sections 5.2 and 5.4).

More recently, alternative approaches have been explored to mitigate these drawbacks. A non-reductive *O*-glycan release method, based on hydroxylamine and 1,8-diazabicyclo(5.4.0)undec-7-ene (DBU), has shown promise for avoiding peeling and improving access to reducing ends.^117^ Although such strategies are not yet widely adopted, they highlight ongoing efforts to overcome the intrinsic challenges of chemical *O*-glycan release.

Overall, chemical methods remain essential for expanding analytical coverage, particularly for *O*-glycans, but they introduce greater variability, artefacts, and increased demands on downstream clean-up.

### 4.3 Workflow formats: in-solution *vs.* solid-phase

Glycan release can be carried out either in-solution or on solid supports, and the choice of format strongly influences yield, reproducibility and tolerance to sample impurities.

In in-solution workflows, proteins are denatured, reduced, alkylated, and enzymatically digested directly in the liquid phase without immobilization. These approaches are straightforward and widely applied, but can be sensitive to matrix components and workflow details. Also, they are frequently applied to complex matrices (*e.g.*, plasma/serum), but salts, detergents, lipids, and other co-extracted components can inhibit enzymatic release or suppress downstream detection if not adequately controlled/removed. Moreover, although many modern in-solution protocols are performed in a one-pot format with minimal transfers, in diluted samples, significant losses can occur as glycoproteins may absorb on container surfaces, including tubes and pipette tips, during sample handling and cleanup steps (*e.g.*, buffer exchange/desalting). These limitations make in-solution methods less suitable for precious or low-abundance clinical material.^118^

In solid-phase approaches, such as immobilization on polyvinylidene fluoride (PVDF)^119,120^ membranes, proteins are physically immobilized on a solid support prior to processing. Once immobilized, all bound protein can be subjected to denaturation, reduction, alkylation, and enzymatic release while remaining on the PVDF membrane. Extensive washing steps can be applied to efficiently remove contaminants, such as salts and detergents, thereby improving the reproducibility and sensitivity of glycan analysis. An additional advantage is that immobilization concentrates analytes at a single location, reducing sample loss and preventing adsorption on container surfaces. However, some losses may still occur due to nonspecific retention on the membrane/support and incomplete release, *i.e.*, immobilization reduces transfer-associated losses but does not eliminate adsorption entirely. This makes solid-phase approaches particularly effective for trace or low-concentration samples where material losses in-solution would otherwise be problematic, and for clinical samples, where background interference can obscure low-abundance glycans.

Overall, in-solution workflows remain attractive when simplicity, speed, and scalability are prioritized, and one-pot implementations can minimize handling losses. For many routine applications, particularly in biopharma glycan profiling, the simplicity of in-solution release outweighs the risk of modest sample losses and matrix-related variability. By contrast, for low-abundance, interference-rich, or otherwise challenging clinical samples, PVDF immobilization offers advantages by enabling extensive washing and reducing transfer-associated losses, thereby improving robustness and reproducibility.

### 4.4 Implications for labelling

The choice of release method directly influences the success of the downstream derivatization and analytical performance, including CE-based separations. Enzymatic release typically produces clean, intact reducing ends with preserved labile modifications, supporting efficient derivatization by reductive amination or newer rapid labelling chemistries (see Section 5). This leads to high labeling yields, consistent mass/charge modification, and reproducible migration behavior in CE as well as reliable quantitative responses in LC-MS and MALDI-based workflows.

By contrast, chemical release can compromise derivatization. Reductive β-elimination often generates peeling artifacts and shortened glycans, lowering labeling efficiency and complicating downstream separations and quantitation. Hydrazinolysis, while better at preserving reducing ends, can introduce chemical adducts that require additional cleanup and may reduce labeling yields. Such artifacts can decrease sensitivity and increase chemical background noise across glycomics platforms and may complicate MS and MS/MS interpretation through additional adducts, neutral losses, or altered fragmentation patterns. Thus, careful selection of the release strategy is critical: enzymatic methods provide the most reliable foundation for quantitative profiling workflows, whereas chemical methods expand coverage, particularly for *O*-glycans, at the cost of increased variability and a greater need for cleanup and method optimization.

## 5. Labelling strategies

Glycan labelling is a cornerstone of glycomics workflows because unmodified released glycans are analytically challenging; they lack intrinsic chromophores for optical detection, are highly hydrophilic, and ionize inefficiently in typical MS ionization methods (ESI, MALDI).^121,122^ These properties make their direct quantification and structural analysis difficult, especially at low concentrations. Labelling strategies overcome these limitations by covalently attaching a chemical tag, most commonly at the reducing end, that improves ionization efficiency by providing enhanced detectability and separation performance and provides more informative fragmentation for structural elucidation across analytical platforms.

For CE-LIF, fluorescent labels primarily enhance optical detectability, enabling highly sensitive and reproducible fluorescence-based detection and supporting absolute quantitation of glycans even at low abundance.^123^ Fluorescent, often highly charged, labels additionally improve electrophoretic resolution, making them particularly suited for quantitative CE-based profiling.^22^ In contrast, reagents applied in CE-MS improve the physicochemical properties of glycans: MS-compatible tags improve ionization efficiency, standardize charge distribution,^22^ and improve isomer separation. Notably, linkage-specific derivatization of sialic acids such as dimethylamidation of α2,6-linked sialic acids combined with lactonization or amidation of α2,3-linked sialic acids^74,124,125^ enables the distinction of these biologically critical linkage isomers by generating unique mass differences detectable by MS.^126^ In addition, these tags significantly influence fragmentation pathways, enabling more reliable structural elucidation and increased sensitivity. Labels that promote controlled fragmentation can enhance linkage and branching information, whereas more stabilizing labels tend to simplify fragmentation towards specific ion series, which can be exploited to selectively obtain certain aspects of glycan structures such as the core composition or monosaccharide sequence. Thus, optimal label design differs depending on whether fluorescence quantification or MS-based structural characterization and sensitivity improvement is prioritized. Hydrophobic or MS-optimized labels may further improve MS sensitivity, although they can alter the electrophoretic migration behavior. Consequently, optimal label selection depends on the primary analytical objective, quantitative profiling or structural characterization, as well as compatibility with the chosen separation and detection platform. In all cases, labelling reduces variability, increases peak sharpness, and stabilizes glycan signals across replicate analysis.^22,127^ To guide label selection throughout this review, we summarize six practical design criteria for reducing-end glycan labels (Table 2).

**Table 2:** Practical aspects for effective glycan labeling reagents

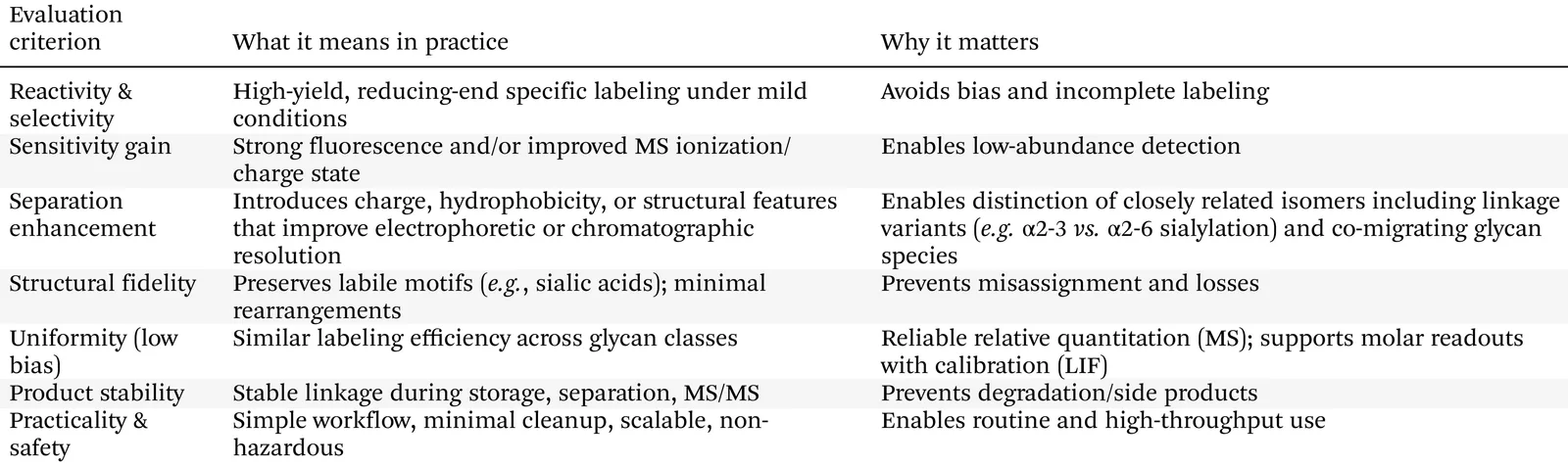

Beyond sensitivity, labeling changes the glycan's charge and hydrophobicity, which directly affects separation.^22,123^ By introducing aromatic or nonpolar motifs, labels improve reverse phase-liquid chromatography (RP-LC) retention, reduce peak tailing, and can enhance CE resolution,^5,128^ supporting discrimination of closely related isomers in complex mixtures. Label-driven increases in hydrophobicity may also improve electrospray efficiency through better desolvation and reduced ion suppression, although the magnitude of this effect depends on the label's ionizable functionality and the sample matrix.^129^

Reproducibility is another major benefit. Labelling reactions such as reductive amination form stable covalent linkages, ensuring uniform modification across molecules and samples.^130^ This consistency leads to reproducible migration times, peak shapes, and signal intensities, which are critical for comparative glycomics studies.

**Fig. 4:**
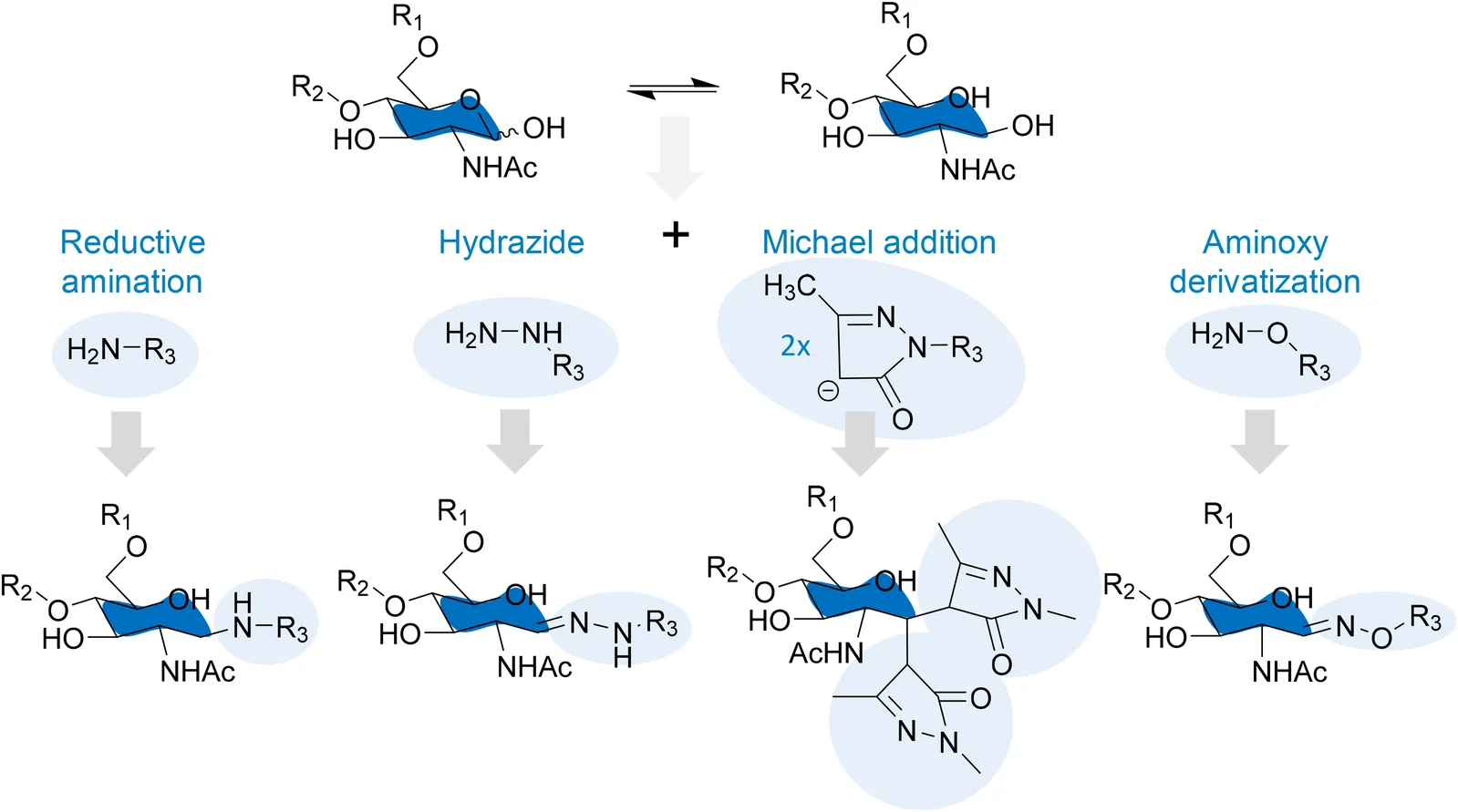
Carbonyl-reactive strategies for reducing-end glycan labeling. Schematic overview of common chemistries used to derivatize released glycans at the reducing end, including reductive amination, hydrazide (hydrazone formation), Michael-addition-based labeling, and aminooxy (oxime) derivatization. The reducing end is shown in its open-chain aldehyde form (reactive carbonyl) and the corresponding cyclic hemiacetal form. R_1_ and R_2_ indicate glycan structural positions: R_1_ denotes the position that may carry core fucosylation (when present), and R_2_ denotes positions extended by additional monosaccharide residues during glycan elaboration. R_3_ represents the attached label functionality (*e.g.*, fluorophore, hydrophobic tag, affinity handle, stable-isotope tag, or permanent charge group for MS).

Taken together, labelling strategies not only extend the sensitivity and scope of glycan analysis but also underpin the CE/LC resolution, separation efficiency, reproducibility, and comparability of CE-based glycomics. A wide range of reducing-end labelling chemistries have been developed, including reductive amination, hydrazide, and aminooxy reactions (Fig. 4), each with distinct trade-offs in speed, stability, labelling efficiency, and analytical performance. Each offers distinct advantages and trade-offs, which are critically assessed in the following subsections. A comparative summary of the major derivatization strategies discussed in this section, including their typical performance trade-offs and platform compatibility (LC, CE, and MALDI-MS), is provided in Table 3.

**Table 3:** Comparative overview of common glycan derivatization (labeling) strategies and their analytical performance

		Chemistry	Separation time	Isomeric separation	Ionization	Sensitivity	Proteoforms	Sialylation stability	Branching	Fucosylation	LC		CE		MALDI-MS	Remarks	Ref.
											FLD	MS	LIF	MS			
**Label**	2-AB	Reductive amination	+	++	++	++	++	++	++	++	^X^	^X^			^X^	Incomplete removal of excess reagent → clean-up required	131–133
	2-AA		++	++	++	++	+	++	++	++	^X^	^X^				Similar to 2-AB but slightly faster migration	131, 132 and 134
	ProcA		+++	++	+++	+++	+++	+++	++	++		^X^		^X^		Strong MS signal, widely adopted	135
	APTS		+++	+++	+	+++	++	++	+++	+++			^X^	^X^		Gold standard in CE-LIF; charged fluorophore	136
	ANTS		+	++	+	++	++	++	++	++			^X^			Less sensitive than APTS	22 and 137–144
	PMP/NMP	Michael addition	++	+	++	++	++	+++	++	++	^X^	^X^				Two tags per glycan; strong UV absorbance; not used in CE-LIF	22, 79 and 145
	GirP/GirT	Hydrazide	+	++	+++	++	+++	++	++	++		^X^		^X^	^X^	Permanent positive charge improves CE-MS	146–152
	**Cascade Blue**		++	+++	++	++	+++	+++	+++	+++			^X^			Fluorescent hydrazide; purification-free MALDI possible	153
	**BODIPY**		++	++	++	++	+	+++	++	++	^X^	^X^		^X^		Bright fluorophore; dual FLD/MS detection	154
	**INLIGHT**		++	++	++	++	++	++	++	++		^X^		^X*^		Commercial isotope labelling kit for quantitative glycomics	155
	**Permethylation**		+++	++	+++	+++	++	+++	++	++		^X^			^X^	Multistep, time-consuming; stabilizes sialylation	156–158 and 159–164
	**Instant AB/PC/**RF-MS		+++	+++	+++	+++	+++	+++	+++	+++	^X^	^X^				Rapid labelling; requires immediate use after release	165–170
	**TMT/AO-TMT**		++	++	++	++	+++	++	++	++		^X^				Multiplexed quantitation; limited glycan adoption	79, 171 and 172–174

### 5.1 Reductive amination-based labelling

Reductive amination remains the most widely applied glycan labelling strategy and serves as the benchmark for quantitative glycomics. The reaction involves condensation of a primary amine with the aldehyde group at the glycan reducing end, forming a Schiff base that is stabilized by reduction to yield a secondary amine, making the product highly stable and resistant to hydrolysis. This stability underpins its widespread use in comparative glycomics. This chemistry results in stoichiometric labelling (one tag per glycan), ensuring accurate quantitation. In practice, the reaction is typically carried out in dimethyl sulfoxide (DMSO) with acetic acid, using sodium cyanoborohydride (NaBH_3_CN) as the reducing agent.^175–177^ While highly effective and robust, this chemistry requires relatively long incubation times (1–2 h at 50–90 °C or 16 h at 37 °C) and involves the use of toxic reagents.^178^

**Fig. 5:**
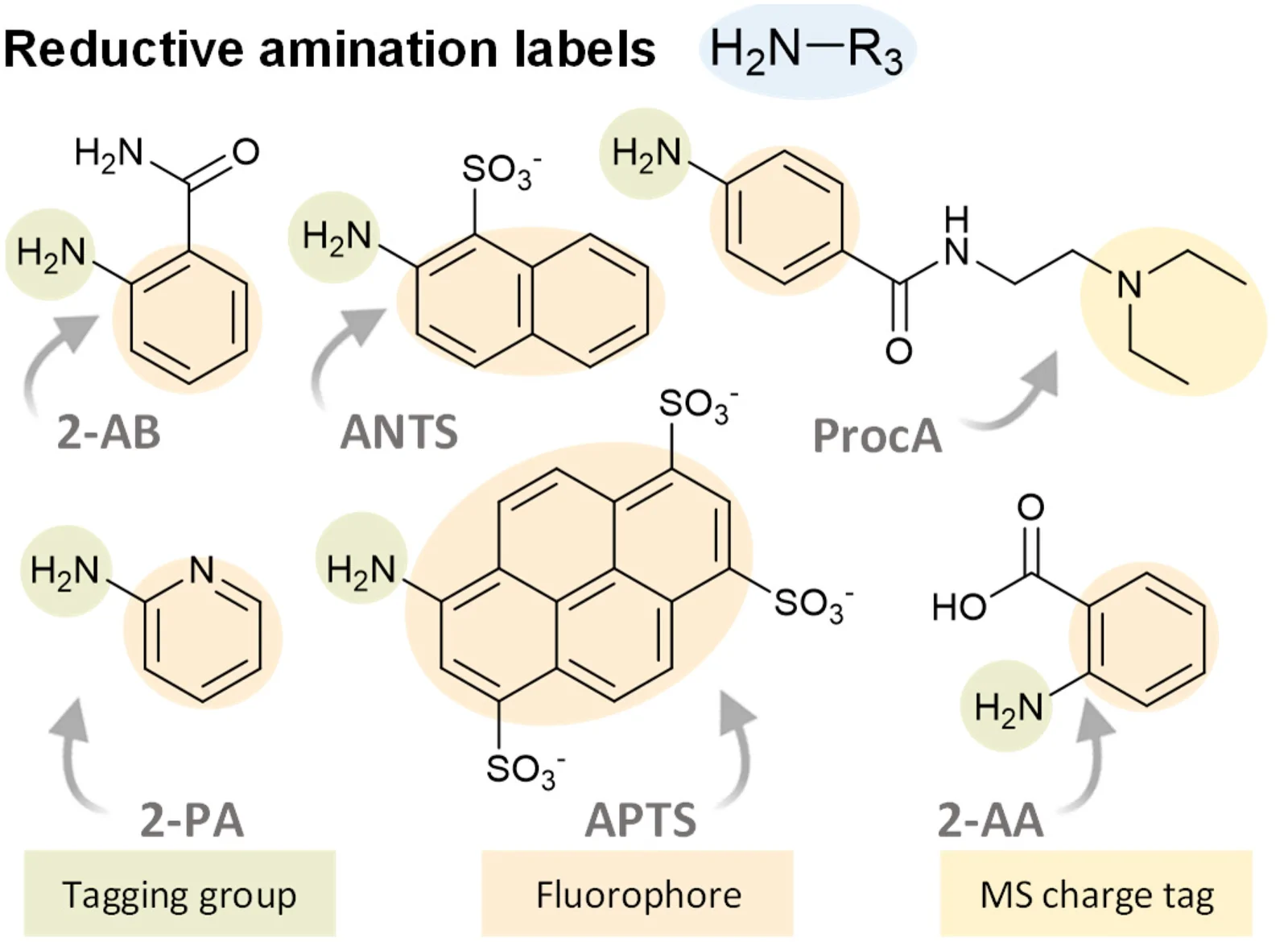
Representative reductive amination labels for glycan analysis and their functional modules. Common reductive amination reagents are shown with their dominant functional groups, including aromatic amines used widely for fluorescence-based detection (*e.g.*, 2-AB, 2-AA) and fluorescent dyes optimized for LIF readout (e.g., ANTS, APTS). Notably, APTS carries three sulfonate groups, imparting a strong net negative charge that promotes efficient separation in reversed-polarity CE-LIF/MS. ProcA contains an MS-enhancing basic groups, a tertiary amine that is readily protonated and typically improves electrospray ionization efficiency and charge-state yield in positive-ionization mode. The choice of label architecture (tagging group, fluorophore, and/or ionizable functionality) influences sensitivity and platform compatibility across CE-LIF, LC-FLD, and CE/LC-MS workflows. Reaction concept is shown in Fig. 3.

Common reductive amination reagents (Fig. 5) include 2-aminobenzamide (2-AB), 2-aminobenzoic acid (2-AA) and 2-amino pyridine (PA), which are widely used in LC-FLD due to their strong fluorescence and reproducibility.^179,180^ In CE-based workflows, however, these labels do not provide the strong, well-defined multi-charge state that underpins many high-performance CE-LIF methods: 2-AB is neutral and 2-AA carries only a single pH-dependent negative charge, resulting in weaker and more condition-dependent electrophoretic control than sulfonated dyes. Procainamide (ProcA) improves MS sensitivity compared with 2-AB/2-AA and is increasingly used in LC-MS workflows.^41,181^ In contrast, charged labels such as 9-aminopyrene-1,4,6-trisulfonic acid (APTS) and 8-aminonaphthalene-1,3,6-trisulfonic acid (ANTS) are specifically used in CE-LIF, and can also be employed in CE-MS,^75,182^ where their strong fluorescence and multiple negative charges enable efficient electrokinetic migration and highly reproducible separation.^22^

In LC-based workflows, reductive amination is considered the gold standard for *N*-glycan separation. By covalently tagging the reducing end, it prevents ambiguity between the closed-ring and open-chain forms of monosaccharides, which can otherwise lead to split peaks or tailing peaks due to inconsistent retention. The covalent bond formed during reductive amination ensures uniform modification across samples, producing reliable retention times and peak shapes across multiple runs, which are critical for comparative glycomics studies. The same benefits extend to CE-based workflows, where consistent covalent labelling contributes to reproducible migration times, sharp electrophoretic peaks and robust quantitative analysis across large sample sets.

The strengths of reductive amination include robustness, reproducibility, structural clarity, and broad platform compatibility. Limitations include relatively long reaction times and the traditional requirement for toxic reducing agents such as NaBH_3_CN, although safer and efficient alternatives such as 2-picoline borane have been introduced.^183^ Suboptimal MS performance may also occur with some conventional labels. Representative reductive amination labels are shown in Fig. 5, illustrating how simple aromatic amines (*e.g.* 2-AB, 2-AA), charged fluorophores (APTS, ANTS), and MS-ionization enhancing tags (ProcA) differ in label functionality and platform suitability.

### 5.2 Michael addition

Michael addition labelling modifies the glycan reducing end *via* a base-catalyzed 1,4-conjugate addition and is particularly valued for improving the stability of sialylated glycans during derivatization and subsequent analysis, reducing the risk of sialic acid loss that commonly occurs under alkaline or in-source fragmentation conditions.^22^

The reaction proceeds in two steps. First, the labelling reagent forms a Michael donor species that reacts with the reducing-end carbonyl of the glycan (Michael acceptor). A water molecule is eliminated, creating an α,β-unsaturated carbonyl intermediate, which then undergoes conjugation with a second reagent molecule. This results in a stoichiometry of two labels per glycan and yields a stable C–C bond at the reducing end. Although efficient, the bulky double-tagged products can dominate glycan behavior in downstream separation.^22^

**Fig. 6:**
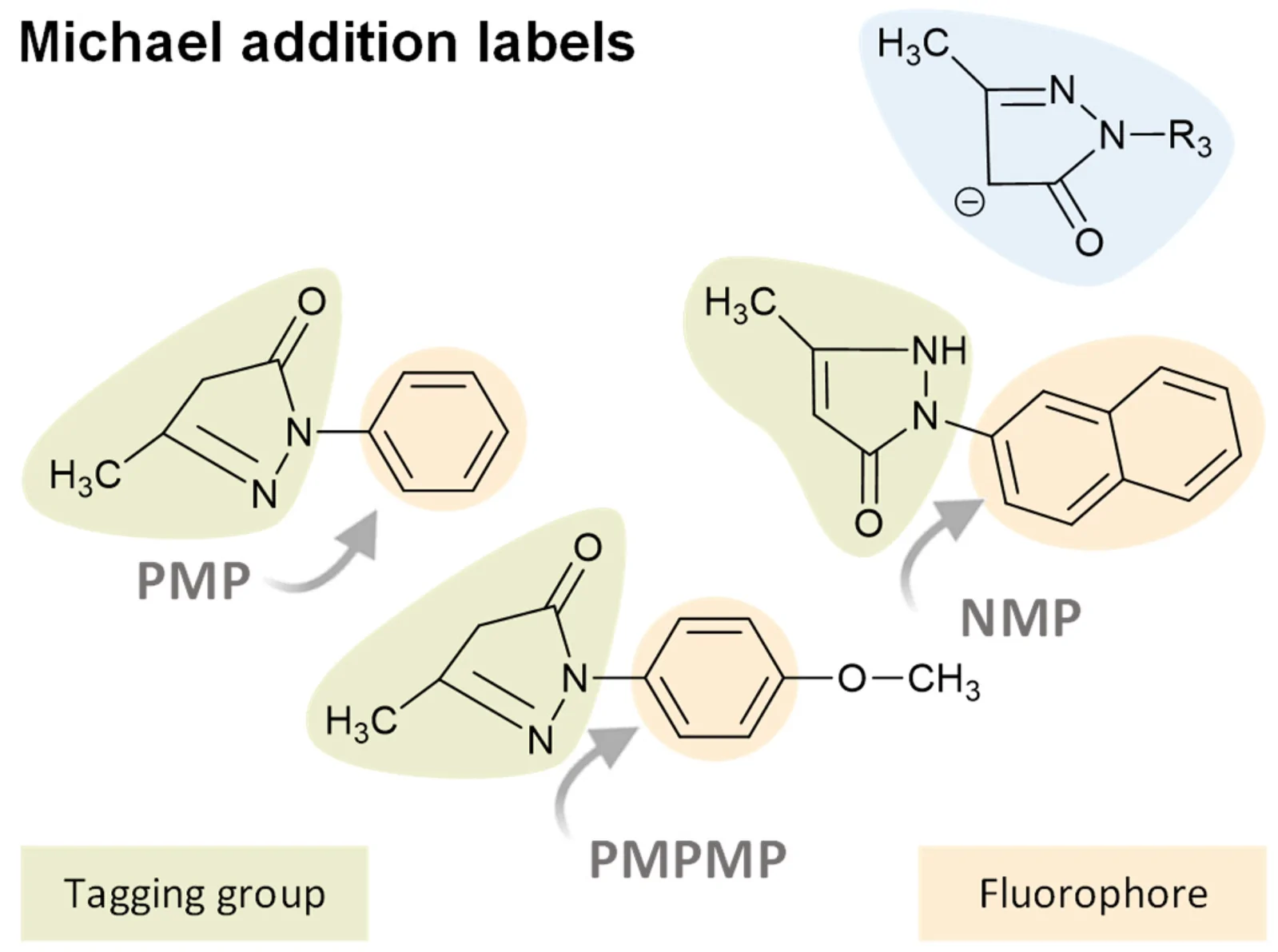
Michael addition-based reducing-end glycan labeling. Schematic illustration of Michael addition labeling in which the reducing end of a released glycan, upon ring opening, reacts with an activated α,β-unsaturated carbonyl (Michael acceptor) to form a stable carbon–carbon bond (reaction concept is shown in Fig. 3). This strategy typically yields rapid, reduction-free conjugation and produces labels that can be tuned by modular substituents. R_3_ denotes the extension of the label.

The most widely used derivatization reagents include (Fig. 6) 1-phenyl-3-methyl-5-pyrazolone (PMP),^184–186^*para*-methoxy PMP (PMPMP)^187,188^ and 1-(2-naphthyl)-3-methyl-pyrazolone (NMP).^22,189,190^ The last of these, introduced by You *et al.*,^191^ offered faster kinetics and improved UV sensitivity compared with PMP. Michael addition has been applied in LC-UV and LC-MS workflows, where the aromatic structures increase hydrophobicity and improve chromatographic retention, while also enhancing ionization efficiency in electrospray ionization (ESI)-MS.^192^

A key advantage of Michael addition is its ability to stabilize sialylated glycans, reducing in-source fragmentation or preparative loss of sialic acids and improving structural fidelity during analysis.^22,42^ PMP-derivatized glycans also introduce a strong chromophore, enabling sensitive UV detection, and the added hydrophobicity improves retention on reversed-phase (C18) columns and enhances peak shape compared to unlabeled native glycans, which cannot be retained on such stationary phases. However, the workflow is more complex than reductive amination, as it requires a double substitution at the reducing end. This also means that glycan's chromatographic behavior is often dominated by the tags, which may reduce the LC resolution of structural isomers.^22,193^ Moreover, no fluorescent variants of pyrazolone reagents have been developed, limiting their compatibility with CE-LIF.

The strengths of Michael addition are the stabilization of sialylated glycans, enhanced UV/MS detection and improved LC separation through added hydrophobicity. The main limitations are the requirement for neutralization and clean-up after derivatization, two tags per glycan can hinder isomer separation, and the lack of fluorescent versions for CE-LIF. Overall, it is less widely adopted in CE and LC compared to standard reductive amination.

### 5.3 Hydrazide labelling

Hydrazide labelling provides an alternative to reductive amination, with the added advantage of introducing permanent charges that enhance MS sensitivity, improve electrophoretic separation in CE, and lead to the possibility of isotopic multiplexing.

Hydrazide reagents react with the aldehyde at the reducing end to form hydrazone linkages, typically under mildly acidic aqueous or mixed solvent (acetic acid, ethanol, water) conditions.^194^ Compared with reductive amination, hydrazide reactions are generally faster due to the higher nucleophilicity of hydrazides, but the resulting hydrazone bond is less stable than the reduced secondary amine generated by reductive amination. This trade-off underlies many of the practical advantages and limitations of hydrazide chemistry.

**Fig. 7:**
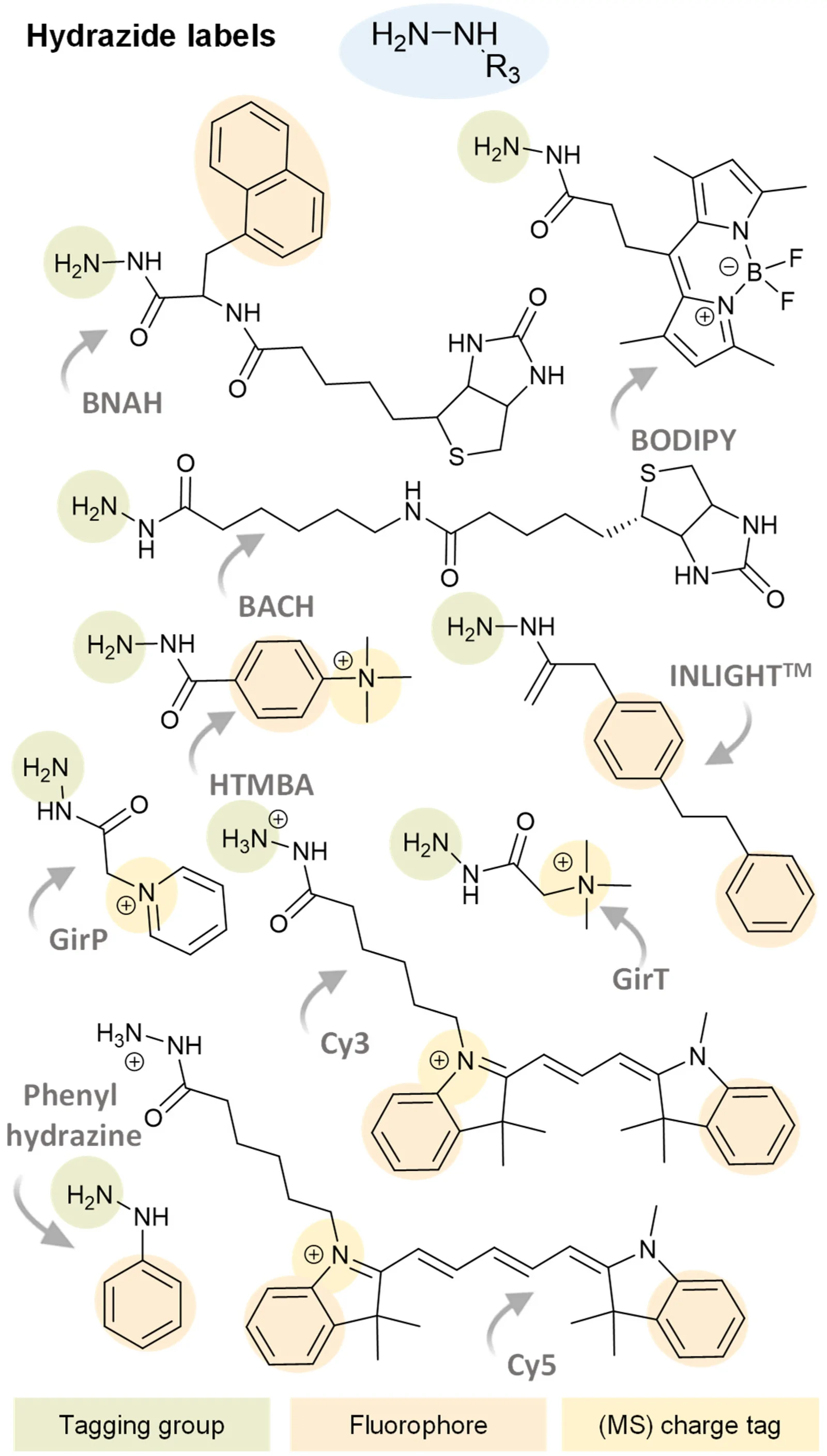
Hydrazide-based reducing-end labeling of released glycans and modular label design. Hydrazide reagents label released glycans at the reducing end *via* hydrazone formation (reaction concept is shown in Fig. 4), typically without requiring a reductive stabilization step. The labeling scaffold can be tuned to incorporate distinct functional modules, including aromatic tagging groups that increase hydrophobicity, fluorophores for optical detection (*e.g.*, BODIPY/Cy dyes), and/or permanent charge tags to enhance electrospray ionization, CE separation and MS sensitivity. For INLIGHT, the aromatic ring can be synthesized with stable-isotope incorporation (*e.g.*, ^13^C) to generate defined mass shifts for multiplexed relative quantification.

Classical hydrazides (Fig. 7) include Girard's T (GirT) and Girard's P (GirP), which introduce permanent positive charges (pyridinium group for GirT and trimethylammonium group for GirP) that improve ionization efficiency in ESI and enhance CE-MS sensitivity.^74^ More specialized reagents include phenyl hydrazine, biotin hydrazide, and Cy3/Cy5 hydrazides for fluorescence or affinity applications.^195–197^ Leteux *et al.*^198^ reported the synthesis of biotin hydrazide derivatives using biotinyl-l-3-(2-naphthyl)-alanine hydrazide (BNAH) and 6-(biotinyl)-aminocaproyl hydrazide (BACH), both of which preserve the closed pyranose form at the reducing end. These were compared with glycans labelled *via* reductive amination using 2-PA. Notably, BNAH integrates two advantageous characteristics; it contains an aromatic chromophore/fluorophore similar to 2-PA, while maintaining the intact pyranose ring of the glycan, as observed with BACH derivatives.^22,198^ Recently, a new cationic hydrazide tag, 4–(hydrazinecarbonyl)–*N*,*N*,*N*–trimethylbenzenaminium (HTMBA), was designed for rapid, high–throughput MALDI–MS glycomics that derivatized glycans efficiently at room temperature in seconds under mildly acidic conditions and gave large signal enhancements without alkali adduct interference, enabling sensitive profiling of complex samples.^194^

Hydrazide labelling can enhance the CE-MS performance by introducing ionizable or permanently charged functionalities that improve electrophoretic behavior and electrospray ionization efficiency, thereby increasing sensitivity. In addition, biotinylated hydrazides enable affinity enrichment on streptavidin linked solid supports,^199,200^ which can reduce matrix interference prior to MS analysis. Consistent with these advantages, a comparative MALDI-TOF-MS study of BNAH, BACH, and related hydrazide tags reported good signal quality and enabled purification-free workflows, although long reaction times remained a practical limitation.^22^ More recently, fluorophore-conjugated hydrazides such as BODIPY hydrazides have provided high fluorescence intensity and sensitivity comparable to those of 2-AB and better MS compatibility.^154^ Hydrazide chemistry also underpins INLIGHT™ labelling, which uses isotopically paired light/heavy reagents for relative multiplexed quantitation of *N*- and *O*-glycans.^155^

The strengths of hydrazide labelling are the enhancement of MS ionization and thus sensitivity, allowing an efficient enrichment step (biotin), purification-free measurements and the possibility of introducing stable isotope labelling (INLIGHT™). The main limitations are that hydrazone bonds are less stable than those obtained through reductive amination and relatively long reaction times are needed.

### 5.4 Permethylation

**Fig. 8:**
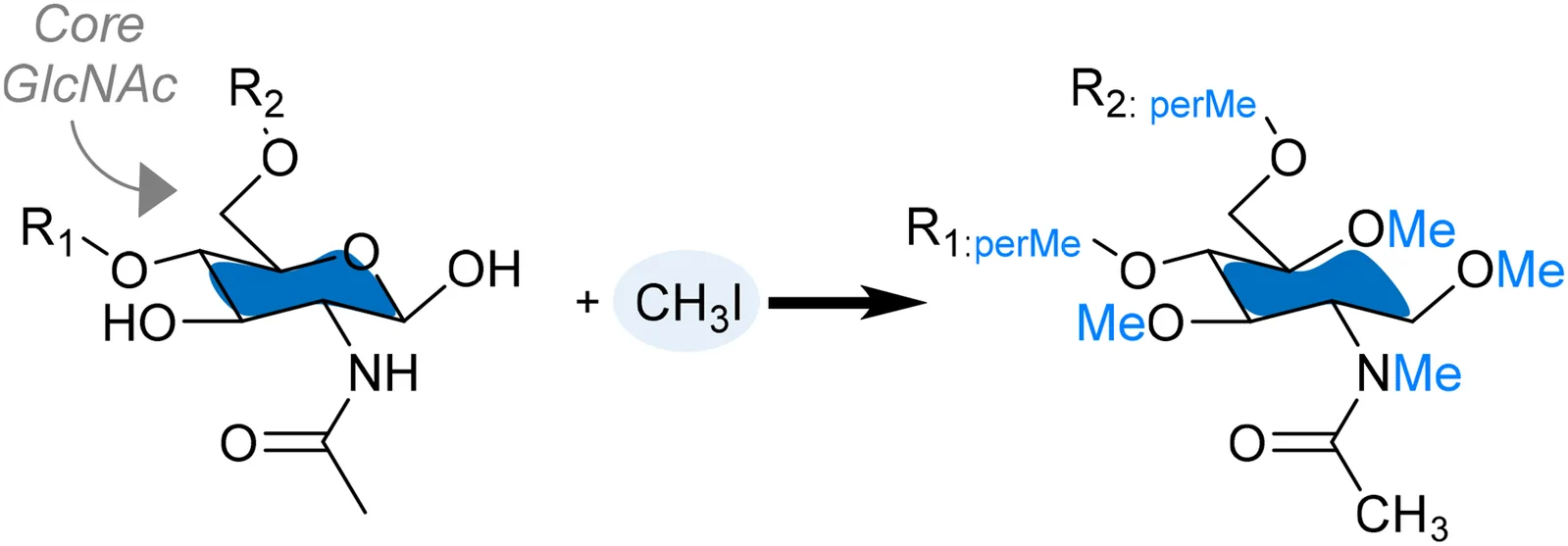
Schematic representation of glycan permethylation for MS analysis. Permethylation replaces labile hydrogen atoms on glycan functional groups with methyl groups, converting hydroxyls into methyl ethers and carboxylates (*e.g.*, sialic acids) into methyl esters. Methylation at the reducing end yields a stabilized, fully methylated glycan with increased hydrophobicity, typically improved ionization efficiency, and often more informative MS/MS fragmentation. Permethylation can also reduce adduct heterogeneity and enhance detection of sialylated species by neutralizing acidic functionalities. Isotopic variants of the methylating reagent (*e.g.*, ^13^CH_3_I or CD_3_I) can be used to introduce defined mass shifts for stable-isotope labeling and relative quantification.

Permethylation is one of the oldest and most widely used chemical derivatization methods in glycomics, particularly in MS-based workflows.^201^ It involves substituting all free hydroxyl and carboxyl hydrogens with methyl groups (Fig. 8), usually using methyl iodide in a strong base such as sodium hydroxide in DMSO.^202^ The reaction increases hydrophobicity, stabilizes labile residues such as sialic acids, and produces derivatives with uniform proton affinities across monosaccharide units^201,202^ that enhance tandem MS fragmentation, including glycosidic and cross-ring cleavages, enabling confident assignment of linkages and branching in structural glycomics analyses.^156,157^ While effective, the method is multistep, requires careful handling of toxic reagents, and often includes extensive clean-up. Moreover, the strongly basic conditions used in permethylation can also induce “peeling”, chemically analogous to the peeling observed during reductive β-elimination (see Section 4.3). Pre-reduction of the reducing end and strictly anhydrous conditions mitigate this issue. Recent advances in solid-phase permethylation have further reduced peeling and sample losses while improving reproducibility.^158^

Permethylation is generally applied in MALDI-TOF-MS and LC-MS workflows,^158,203–205^ where increased hydrophobicity improves chromatographic retention and signal intensity. It is less commonly used in CE-based separations because permethylation neutralizes acidic groups and reduces the overall charge differences between glycoforms, diminishing the strong charge-to-size selectivity that CE typically exploits for glycan separations. As a result, separations can become less robust and more dependent on secondary effects, while the workflow also diverges from standard aqueous CE conditions. Permethylated glycans provide strong positive-mode ESI signals and yield extensive structural information upon fragmentation. In tandem MS, permethylation enhances both glycosidic and cross-ring fragmentation, facilitating the determination of branching patterns, linkage positions, and isomeric configurations (*e.g.* configurational and conformational). This makes permethylation highly valuable for detailed structural glycomics, though less suited to high-throughput profiling.

The strengths of permethylation are the stabilization of sialylated and other labile residues, enhancement of ionization and detailed fragmentation patterns, enabling detailed structural assignment including branching and linkages. The main limitations are considered to be the multistep and time-consuming labeling, the use of toxic reagents, the risk of oxidative degradation and peeling, high sample losses in solution-phase workflows and not being applicable to CE-based methods.

### 5.5 Isotopic and isobaric labelling

Isotopic and isobaric strategies extend glycan derivatization beyond detection, enabling quantitative and multiplexed workflows for comparing glycan abundances across multiple samples. While widely established in proteomics,^206–210^ their use in glycomics remains an emerging but rapidly developing area.

**Fig. 9:**
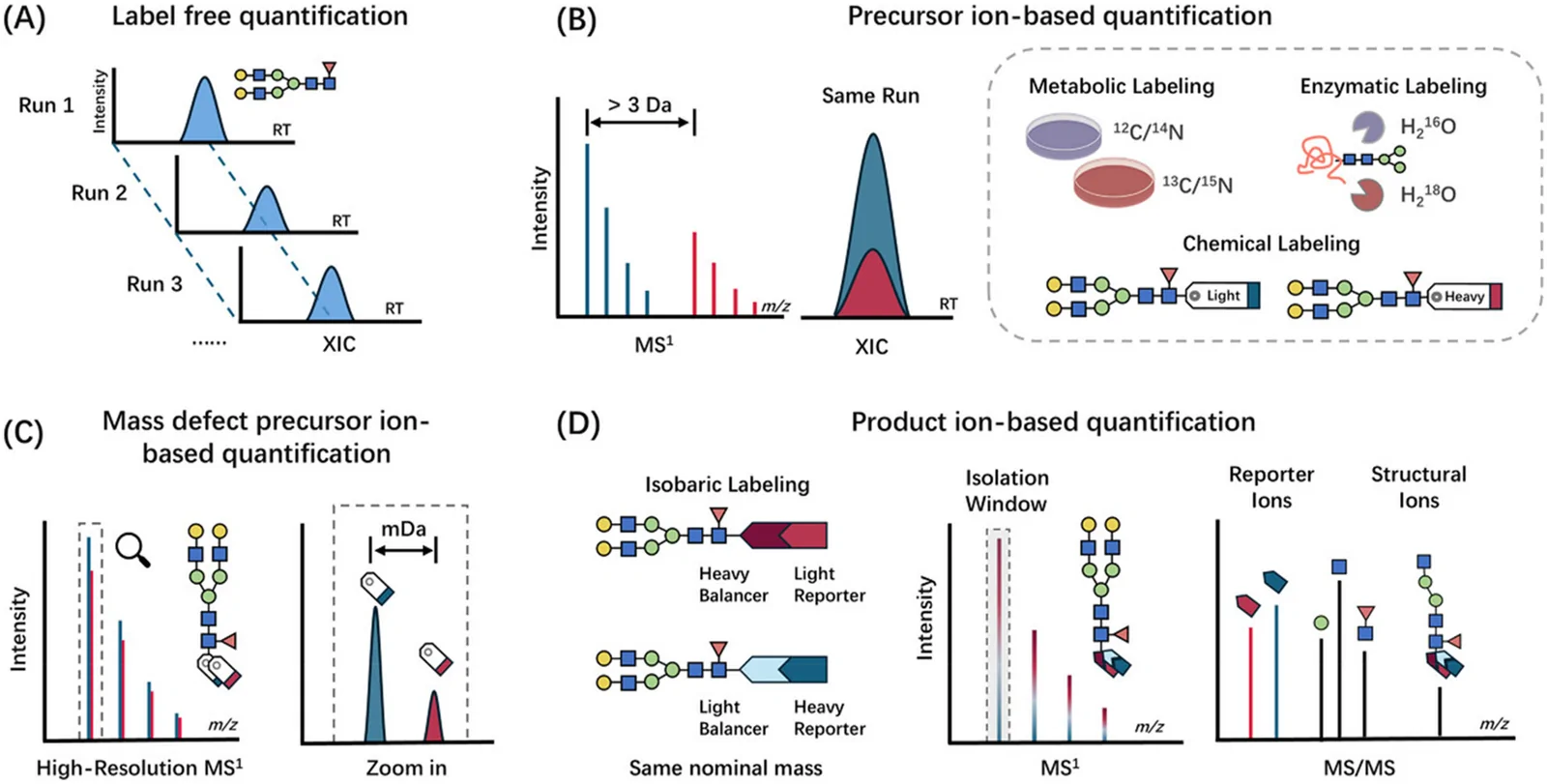
Overview of quantitative strategies for released-glycan glycomics. (A) Label-free quantification compares extracted ion chromatograms (XICs) across separate runs and relies on robust alignment and normalization to control run-to-run variability. (B) Precursor ion-based isotope labeling introduces defined mass offsets between “light” and “heavy” forms that co-elute and are quantified at the MS1 level; labeling can be achieved through metabolic incorporation, glycosyltransferase-mediated enzymatic labeling, or chemical derivatization. (C) Mass-defect precursor-ion quantification uses small mDa-scale differences and therefore requires high-resolution MS^1^ to resolve labeled pairs and reduce spectral complexity. (D) Product ion-based quantification uses isobaric tags that are indistinguishable at MS^1^ but yield channel-specific reporter ions upon MS/MS, enabling multiplexed quantification while glycan fragments support structural assignment. Reprinted with permission from Wang, Zhang and Li (Scheme 1).^127^

Fig. 9 provides an overview of these quantitative concepts, spanning label-free comparison across runs (Fig. 9A), precursor-ion-based isotope strategies (Fig. 9B and C) and product-ion-based isobaric tagging (Fig. 9D).

In isotopic labelling (Fig. 9B), “light” and “heavy” reagents incorporate defined numbers of stable isotopes (^13^C, ^18^O and ^15^N),^211^ generating a precursor mass offset while typically maintaining co-elution/migration and enabling relative quantitation from MS1 signals.^212–215^ A related variant is mass-defect labeling, which uses small mDa-scale shifts resolvable only by high-resolution MS1 enabling resolving precursor ions (Fig. 9C).^127^ In isobaric labelling (Fig. 9D), channels are mass-balanced to appear identical at the precursor level but release distinct reporter ions upon fragmentation, allowing multiplexed quantitation.^127,216–219^ Stable isotope labelling can be achieved in three ways, metabolic, enzymatic or chemical labelling.

#### 5.5.1 Metabolic labelling

Metabolic labelling^220,221^ introduces isotopically modified monosaccharides (*e.g.* mannosamine, galactosamine, fucose, and sialic acid) that are incorporated into glycans through endogenous cellular biosynthetic pathways. This powerful *in vivo* approach enables monitoring of glycan turnover, biosynthetic pathway flux, and dynamic regulation of glycan synthesis in cell culture, animal models and human studies.^222,223^ However, metabolic labelling has limited applicability to released glycan profiling because incorporation is often incomplete, cell-type specific and dependent on metabolic accessibility.^224–227^ In glycoproteomics, SILAC (stable isotope labelling by amino acids in cell culture) remains the most established metabolic isotope method, allowing the comparison of differentially labelled cell populations to study changes in glycopeptides under varying biological conditions.^228^ A related approach, isotope-targeted glycoproteomics (IsoTaG), uses metabolic incorporation of chemically modified sugars into cellular glycans, followed by click chemistry attachment of isotopic biotin tags, enabling selective enrichment and intact glycopeptide profiling by MS.^229^

#### 5.5.2 Glycosyltransferase-mediated labelling

Enzymatic labelling^230,231^ utilizes specific glycosyltransferases to transfer isotopically labelled monosaccharides onto well-defined acceptor sites *in vitro*, offering excellent control over labelling sites and reaction conditions and allowing the study of biosynthesis related dynamics downstream of the glycotransferase consuming the isotopically labelled monosaccharides. For instance, a sialyltransferase can transfer a CMP-^13^C-sialic acid onto a terminal galactose residues, while fucosyltransferases incorporate ^13^C-fucose. These reactions offer excellent selectivity, specificity and preserve the native glycan structure. Despite its precision, enzymatic labelling is constrained by the availability of donor substrates, competition with endogenous unlabeled substrate pools that dilute the isotopic label and complicate absolute flux detection, high reagent costs, and limited throughput.^127,225,232^ Its greatest value lies in targeted enrichment where enzymatic incorporation of an isotopically labeled monosaccharide is used to confirm the identity, linkage, or modification of a specific glycan, rather than for large-scale quantitation of all glycans.

Enzymatic labelling is especially useful for enhancing the detection sensitivity in MS, since the addition of a labelled residue can provide a distinct mass shift, facilitating identification and quantification of labelled glycans.

#### 5.5.3 Chemical labelling

Chemical labelling^233,234^ remains the most widely applied method in glycomics; it involves the covalent attachment of isotope-containing tags to glycans, either through derivatization of released glycans or at specific functional groups. Reductive amination is a widely used approach, where isotopically labelled amines, such as ^13^C_6_-aniline or ^12^C/^13^C-pyridine derivatives, are conjugated to the reducing end of glycans. Another popular method is permethylation using isotopically labelled methyl iodide (CD_3_I), which not only labels the glycans but also improves their ionization efficiency in MS.^212,235^ However, a known limitation of deuterium-based labels in LC workflows is that deuterated glycans exhibit slightly earlier elution times than their non-deuterated counterparts on reversed-phase columns.^236,237^ Hydrazide-based INLIGHT™ uses isotopically paired hydrazides (light/heavy) and has been commercialized, offering relative quantitation of *N*- and *O*-glycans with minimal sample preparation.

#### 5.5.4 Isobaric labelling

**Fig. 10:**
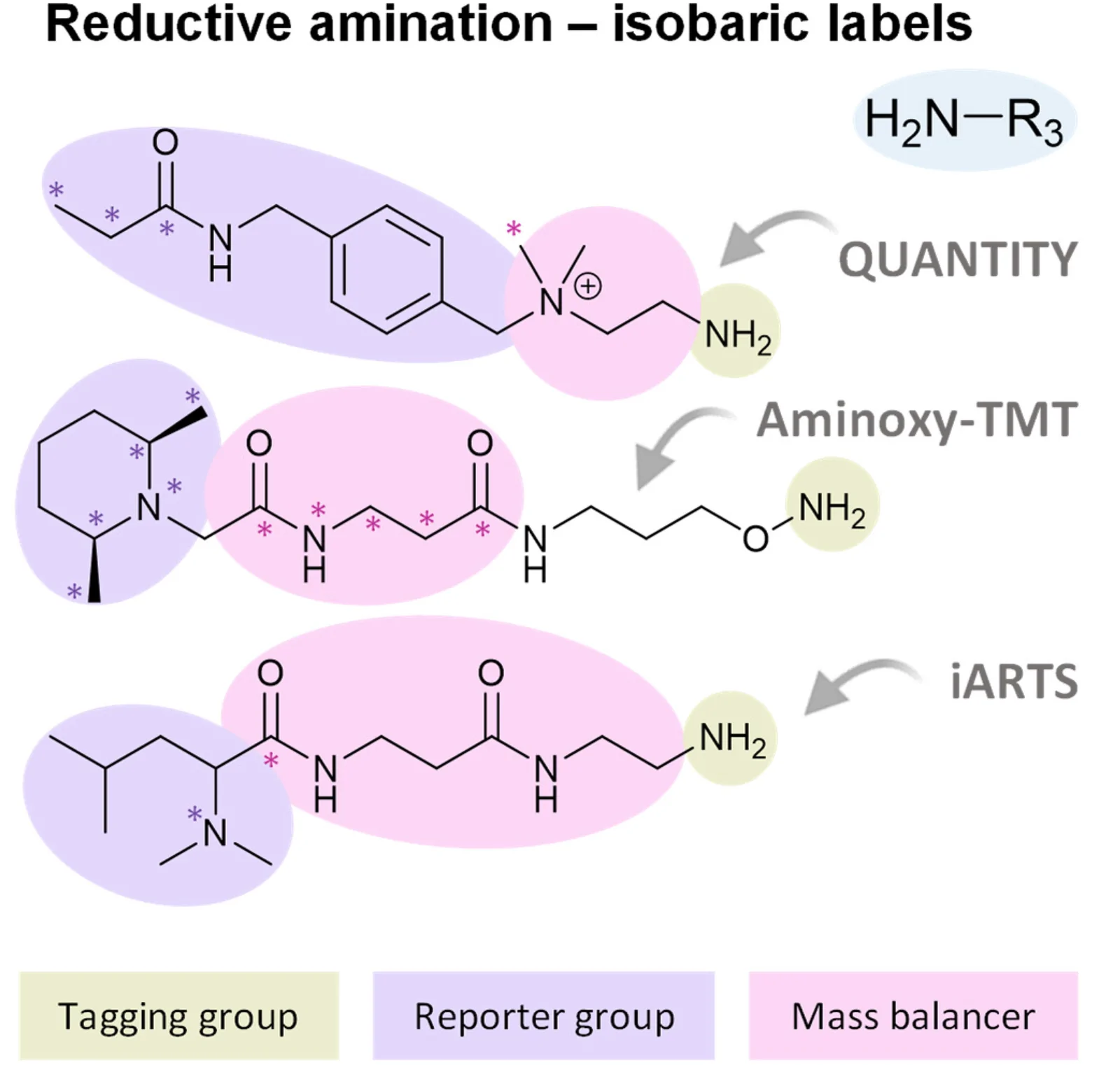
Modular architecture of isobaric tags used for MS/MS-based glycan quantification. Schematic comparison of representative isobaric labeling chemistries (QUANTITY, aminooxy-TMT, and iARTs), highlighting the three functional elements of these reagents: a carbonyl-reactive tagging group for covalent attachment to glycans (R_3_), a reporter group that generates sample-specific ions upon MS/MS, and a mass balancer that maintains identical precursor masses across channels, but remains undetected during fragmentation. Multiplexing is achieved by distributing stable isotopes between the reporter and balancer modules (indicated by *). In QUANTITY, four channels can be created by combining alternative isotope compositions in the balancer (unlabeled, ^13^C-labeled, and ±^2^H variants) with reporter variants (±^13^C). Aminooxy-TMT enables higher-plex quantification (commonly 6-plex) through isotope encoding in the reporter/balancer system, while iARTs provides a 2-plex implementation (*e.g.*, ^13^C *vs.*^15^N encoding).

Isobaric chemical tags were originally developed for peptide quantification in proteomics, as exemplified by tandem mass tags (TMT) and iTRAQ,^238^ and the same modular design has been adapted for glycomics through glycan-reactive derivatives (*e.g.* aminooxy-TMT).^171,218,239^ Additional glycan-oriented implementation includes iARTs^238^ (a 2-plex isobaric tag) and QUANTITY,^218^ which achieve multiplexing by distributing stable isotopes between the reporter and mass balancer modules (Fig. 10). These tags share a modular structure composed of three key components: a mass reporter group that allows sample specific quantification of target compounds, a mass normalizer (or balancer) group that cleaves off as neutral loss remaining undetected by MS, and a reactive functional group for covalent attachment to target molecules. In glycomics, attachment is typically achieved through carbonyl-reactive functionalities (*e.g.*, aminooxy or hydrazide groups) that couple to the reducing end of released glycans, forming stable oxime or hydrazone linkages. Upon MS/MS fragmentation, the tag releases channel-specific reporter ions, enabling relative quantification of multiple samples in a single run, while the glycan-derived fragments support structural assignment. Beyond multiplexing, isobaric tags can boost the signal for very small sample amounts, as demonstrated in TMT-based SCOPE-2 (single cell proteomics by mass spectrometry, second generation) single-cell proteomics,^240^ highlighting their potential for single-cell glycomics applications. It should be noted, however, that TMT and related isobaric reagents are currently limited to DDA acquisition settings, as reporter ions lose their quantification capability under DIA conditions due to co-fragmentation of co-isolated precursors. Although conceptually powerful, isobaric labelling of glycans remains less common than in proteomics, with only a few studies published in the literature.^218^ While challenges such as ratio compression, isotope impurity correction, and loading normalization are shared with proteomics applications where isobaric tags remain widely used, glycan-specific limitations further hinder broader adoption: glycosidic bonds are more labile than peptide bonds and tend to fragment before reporter ions are generated, resulting in inefficient and poorly reproducible reporter ion yields; dedicated glycan-reactive isobaric reagents remain scarce; and the structural complexity of glycans makes channel cross-talk arising from co-isolation of isobaric precursors particularly difficult to resolve.

#### 5.5.5 Isobaric quantitative glycomics using stable-isotope labeled internal standards

Stable isotope-labelled standards have emerged as valuable tools for improving the accuracy and comparability of quantitative glycomics workflows. By spiking isotope-labelled glycans or glycopeptides into samples at known concentrations, variations arising from sample preparation, derivatization, ionization, and instrument performance can be minimized, enabling both relative and absolute quantification. Echeverria *et al.* demonstrated the use of ^13^C-labelled complex *N*-glycans as internal standards for absolute glycan quantification, providing improved analytical accuracy and traceability.^241^ Similarly, Zhou *et al.* reported the use of iGlycoMab stable isotope-labelled glycans as internal standards for reliable LC-MS quantitative glycomics, demonstrating improved normalization and quantitative accuracy across glycomics workflows.^242^ More recently, stable isotope-labelled glycopeptide standards enabled accurate absolute quantification of IgG glycoforms and subclass-specific glycosylation, as demonstrated by Li *et al.*^243^ Sanda *et al.*^244^ further employed a stable isotope-labelled IgG1 Fc glycopeptide standard for LC-MS/MS quantification of IgG glycoforms, improving analytical precision and robustness. A recent review by Yun *et al.* summarized the growing range of isotope-labelling strategies available for quantitative glycomics and highlighted their potential for standardization and inter-laboratory harmonization.^214^ Although these approaches have primarily been demonstrated using LC-MS and MALDI-MS workflows, the same principles are directly applicable to CE-MS and may facilitate improved quantitative reproducibility and absolute quantification in future CE-based glycomics studies.

#### 5.5.6 Practical implications for CE- and CE-MS based workflows

Isotopic approaches (*e.g.* INLIGHT, ^13^C-paired reductive amination) are well suited to precise relative quantitation of a small number of samples. Isobaric methods (*e.g.* aminooxy-TMT) provide higher throughput and multiplexing capability but at the cost of workflow complexity and increased instrument demands. Both strategies remain MS-centric, with minimal implementation in CE-based workflows to date, although hydrazide isotopic tags could, in principle, be adapted for CE-MS.

The strength of isotopic and isobaric labelling is the ability to enable relative and multiplexed quantitation. Isotopic tags are robust and chemically simple and isobaric tags enable high-throughput multiplexing.

In regard to limitations, these tags have limited CE compatibility (lack of charge), higher cost and are relatively complex. There is a lack of standardized protocols, unlike classical fluorescent or reductive amination labels where established reaction conditions, reference standards, and purification steps ensure reproducibility, and in general there is a lower adoption in glycomics compared with proteomics.

### 5.6 Effects of labelling on MS fragmentation behavior

The choice of labelling strategy in glycomics plays a crucial role in shaping MS fragmentation behavior, ionization efficiency, and ultimately the ability to extract structural information from mass spectra.

#### 5.6.1 Fragmentation mechanisms and influence of charge localization

**Fig. 11:**
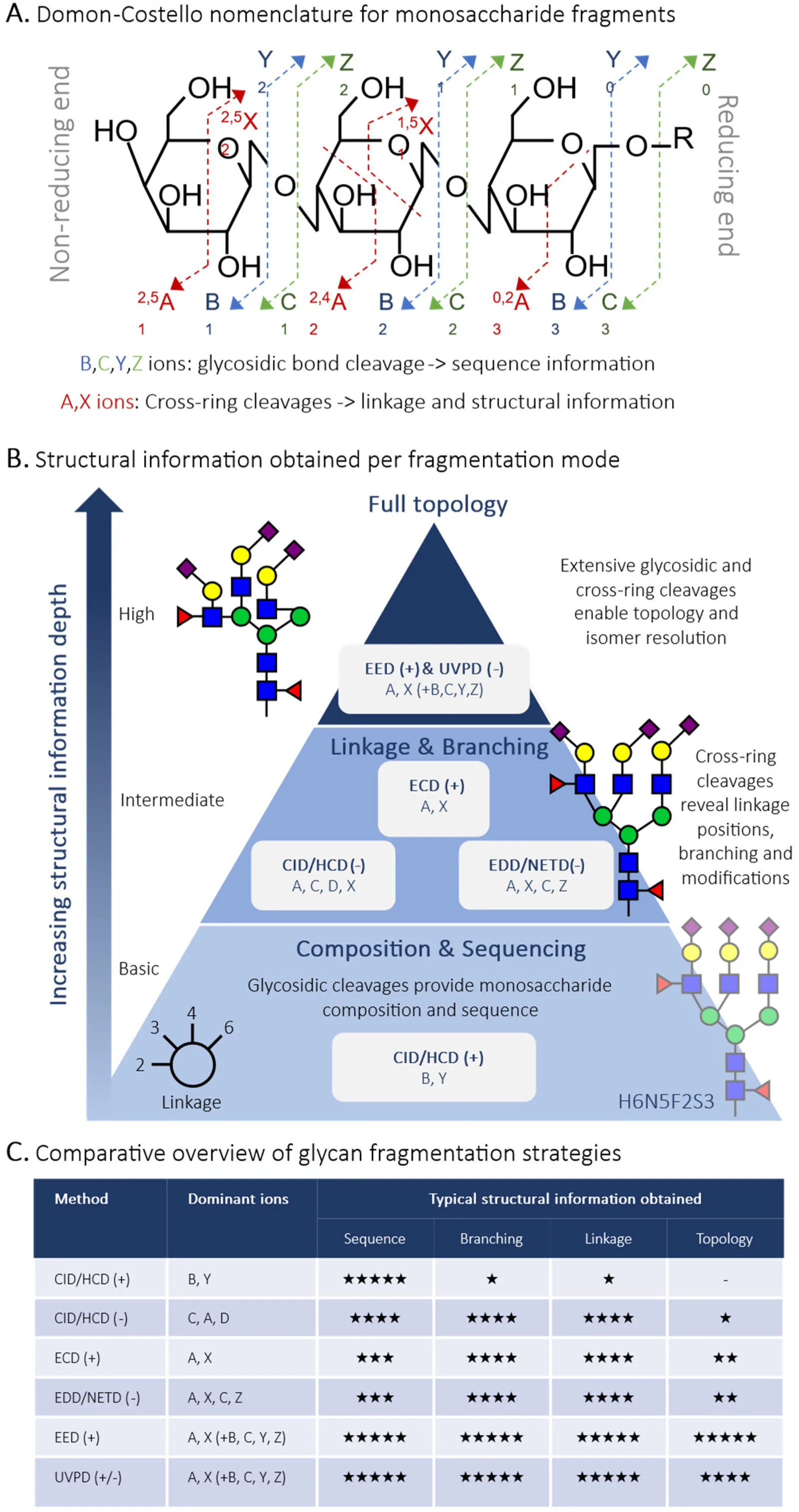
Structural information hierarchy accessible to MS/MS fragmentation methods in glycomics, illustrated for a complex-type *N*-glycan (H6N5F2S3). (A) Domon–Costello nomenclature for glycan fragment ions on a generic chain (non-reducing end left, reducing end right). Glycosidic cleavages (B/C, Y/Z, blue) retain the non-reducing- or reducing-end portion of the glycan. Cross-ring cleavages (A, X, red) break within a sugar ring and, depending on subtype, can encode linkage and branching information. Adapted from Domon and Costello, *Glycoconjugate Journal*, 1988, **5**, 397–409.^245^ (B) Fragmentation methods arranged by depth of structural information obtained, from composition/sequencing (base) to full topology (apex), with polarity and dominant ion types per method. The H6N5F2S3 cartoons show the structure as resolved at each tier; the base-tier cartoon is shown with reduced opacity, as composition is well established at this level but the precise branching and linkage pattern is not directly confirmed. (C) 1–5 star ratings of each method's relative capacity to determine sequence, branching, linkage, and topology, corresponding to the pyramid tiers (★★★★★ = apex-level, comprehensive/low-ambiguity; ★★★ = mid-tier, partial information; ★ = base-tier, minimal or limited to select diagnostic ions; - = no meaningful information). Cross-ring ion subtypes vary in diagnostic value: ^1,5^A/^1,5^X form independently of neighboring linkages and confirm sequence only, while ^2,4^A, ^0,2^A, and D-type ions depend on the substitution pattern and encode linkage/branching. D-ions specifically arise from a combined glycosidic plus cross-ring cleavage at a branch-point residue, making them particularly useful for distinguishing antenna positions (*e.g.* 1–3 *vs.* 1–6 arm). EED(+) assumes permethylated, metal-adducted precursors on electron-capable instruments (FT-ICR/Omnitrap); UVPD(+/−) reflects best-case negative-mode performance, as positive-mode UVPD behaves more like CID/HCD(+).

Tandem MS of glycans primarily produces two fragment types: glycosidic cleavages (B- and Y-ions) that define sequence, and cross-ring cleavages (A- and X-ions) that provide linkage and branching information (Fig. 11A).^245^ Because different activation methods favor these fragment classes to different extents (Fig. 11B), the observed spectra are strongly governed by charge location and mobility.^246^ Under collision-induced dissociation (CID) or higher-energy collisional dissociation (HCD), fragmentation generally proceeds through vibrational energy transfer, favoring glycosidic cleavages, whereas electron-transfer dissociation (ETD) or electron-capture dissociation (ECD) involve electron transfer to multiply charged precursors, producing complementary cross-ring fragments that preserve labile substituents. An important exception is negative-ion mode CID/HCD, which can yield comparatively rich structural information on glycans. For deprotonated precursors, particularly acidic glycans, CID/HCD can produce abundant diagnostic fragments, including cross-ring cleavages (A/X-type ions) and other linkage-informative ions, improving discrimination of positional and linkage isomers even without electron-based dissociation. As a result, negative-mode fragmentation is frequently favored for native or highly acidic glycans, whereas positive-mode strategies rely more strongly on derivatization and charge engineering to achieve comparable sensitivity and interpretability.

Beyond these established methods, a small number of specialized activation techniques extend access to cross-ring information further still (Fig. 11B). The hierarchy of structural information shown in Fig. 11B reflects a general principle of tandem MS: different activation methods provide different depths of structural insight, ranging from molecular composition to complete structural elucidation. Similar concepts are widely applied in metabolomics, where orthogonal fragmentation strategies are combined to move from compound identification towards the localization of functional groups, double bonds, stereochemistry, and molecular topology.^247^ Electronic excitation dissociation (EED) applies an electron–ion interaction conceptually related to ECD, but *via* excitation achieved by higher electron energy rather than capture, typically to permethylated, metal-adducted (Li^+^/Na^+^) glycan ions on FT-ICR or electron-capable Orbitrap/Omnitrap instruments.^248–250^ This produces a near-complete glycosidic (B/C/Y/Z) and cross-ring (A/X) ion ladder spanning the entire structure, currently providing the most comprehensive single-method access to full topology, at the cost of additional derivatization and specialized instrumentation. Ultraviolet photodissociation (UVPD) can achieve comparably broad fragment coverage through direct photon absorption rather than electron-transfer chemistry:^251,252^ in negative-ion mode, UVPD of deprotonated glycans generates an extensive mixture of glycosidic and cross-ring ions across both series, whereas in positive-ion mode it behaves more like CID/HCD, favoring B/Y-type cleavages. For acidic glycans, electron detachment dissociation (EDD) and negative electron transfer dissociation (NETD) extend electron-based activation to multiple negative ions, generating radical-driven C/Z and cross-ring (A/X) fragments while retaining sulfate and sialic acid groups that are prone to loss under positive-ion activation,^253–256^ making these methods particularly suited to glycosaminoglycans and highly sialylated glycans.^256,257^ The experimental foundation for UVPD and EDD/NETD has so far been established largely for glycosaminoglycan and glycosphingolipid systems, with extension to released *N*/*O*-glycans comparatively less validated. Because EED, UVPD, and EDD/NETD derive their cross-ring selectivity from electron- or photon-driven mechanisms rather than from charge-localization effects, they provide more uniform access to linkage and branching information regardless of derivatization strategy, though their broader adoption remains limited by instrument availability relative to CID/HCD.

Reducing-end labels that increase proton affinity (*e.g.* tertiary or quaternary amines such as ProcA) or introduce a fixed positive charge (Girard reagents) tend to localize charge toward the reducing end, improving ionization and generating abundant B/Y-type ions under CID or HCD.^22,41^ Because charge movement is restricted, cross-ring cleavages can be less prominent, though they reappear under ETD/ECD or high-energy conditions where charge mobility increases.^258,259^

Reducing-end labels can also influence isomeric separation in CE and LC and may introduce label-dependent artifacts (*e.g.*, altered adduct patterns or peeling of monosaccharides); this is particularly relevant for acidic and labile glycans, as discussed below.

#### 5.6.2 Impact of label chemistry on acidic and labile glycans

For neutral glycans, reducing-end derivatization largely dictates charge localization and fragmentation patterns. Sialylated, sulfated, and phosphorylated glycans present special challenges because their intrinsic negative charges dominate ionization and fragmentation, independent of any reducing-end tag. These species often exhibit neutral losses such as SO,^3^ phosphate, or sialic acids, reducing linkage information and overall signal intensity. In addition, even neutral oligomannose *N*-glycans can exhibit in-source decay and extensive adduct heterogeneity, which can complicate quantitation and spectral interpretation when ionization or source conditions are not carefully controlled.

Loss of sialic acids is a recurrent problem for both native and labelled glycans. Stabilization can be achieved through esterification or permethylation,^22,53,260^ both of which reduce in-source desialylation by masking acidic functionalities. Esterification is comparatively targeted and can preserve much of the native backbone, but it adds derivatization and cleanup steps that may introduce variability and sample loss.^261^ Permethylation, in contrast, globally modifies hydroxyl and carboxyl groups, increasing hydrophobicity and typically enhancing positive-mode signal intensity and MS/MS interpretability; however, it is also a multistep workflow that requires rigorous cleanup and can be time-consuming, with performance depending on reaction completeness and sample handling. In practice, the choice between esterification and permethylation reflects a trade-off between workflow complexity, robustness, and the desired depth of structural information. Different reducing-end labels also lead to variable adduct formation, charge-state distributions in ESI, and fragment-ion ratios; ionization efficiency is often suppressed for highly sialylated glycans unless acidic functionalities are stabilized by derivatization.^88,260^

#### 5.6.3 Practical implications for CE- and MS-based workflows

Label selection determines ionization polarity and charge-state distribution. Sulfonated fluorophores (*e.g.* APTS, ANTS) generate multiply charged anions for CE-MS in reversed polarity, providing excellent migration reproducibility but the strong negative charge can limit the structural information obtainable from MS/MS.^262^ Amine-containing or permethylated labels promote cation formation in CE-MS or LC-MS, yielding richer fragmentation patterns and improved sensitivity, although methods like PGC–LC–MS/MS can also be used with permethylated glycans for detailed structural analysis. Following permethylation, positive-ion mode remains the dominant approach for extracting linkage and branching information.^73,263^

In practice, highly charged or proton-affinitive labels (procainamide, Girard reagents) are preferred for sequencing and quantitative workflows, while neutral or permethylated derivatives combined with ETD, ECD or EAD provide the most comprehensive linkage and branching information.

Overall, fragmentation outcomes are jointly determined by label chemistry and instrumental parameters, making method optimization essential for each analytical goal. The choice of derivatization strategy directly shapes sensitivity, fragmentation efficiency, structural stability, and isomeric resolution in CE/LC, underscoring the need to align labelling chemistry with the specific analytical objectives of glycomics workflows.

### 5.7 Instant labelling strategies

Recent developments in glycan derivatization have focused on rapid, MS-compatible labelling chemistries that simplify workflows and improve throughput without compromising sensitivity or structural fidelity. These “instant” labels were developed to address two practical bottlenecks of classical reducing-end derivatization, long incubation times and multi-step handling, by enabling fast, reduction-free coupling and integrated detection features. In the following subsections, we first outline the underlying reaction design and linkage chemistry, then summarize the analytical performance and platform compatibility, and finally discuss practical limitations and the current scope.

#### 5.7.1 Chemistry and mechanism

Instant labeling reagents most commonly use activated *N*-hydroxysuccinimide (NHS)-carbamate or amine-reactive urea chemistries to derivatize the reducing end of released glycans within minutes, forming stable carbamate or urea linkages. Unlike reductive amination, which typically requires long incubations and a reduction step, these reactions proceed rapidly under mild conditions and yield stable end-products. A key feature of NHS-carbamate reagents is self-quenching hydrolysis; the by-products generated after reaction are non-fluorescent, minimizing the background signal and enhancing detection limits, which is a valuable property for fluorescence-based detection (FLD or CE-LIF).^41,264,265^

#### 5.7.2 Analytical performance and platform compatibility

Instant labels are designed for dual-mode detection, performing equally well in fluorescence- and MS-based assays.

Reagents such as Waters RapiFluor-MS (RF-MS), InstantPC (IPC), and InstantAB (IAB) combine strong fluorescence with excellent positive-mode ionization efficiency.^41,264,266^ These dual properties enable simultaneous quantitative analysis by CE-LIF or LC-FLD and structural elucidation by CE-MS or LC-MS. When paired with rapid enzymatic *N*-glycan release (*e.g.* PNGase F in 5–10 min), complete sample preparation can be achieved in under 30 min.

Efficient labelling depends on the presence of an open-chain reducing end, which is transient after release; hence, maximum yield is achieved when reagents are added immediately after enzymatic cleavage. If glycans are dried or stored, the reducing end re-cyclizes and the labelling efficiency decreases sharply.

While instant reagents perform exceptionally well for purified glycoproteins or recombinant biopharmaceuticals, complex matrices such as plasma or serum can introduce challenges; interfering proteins, salts, and free amines may compete for the reactive groups or quench fluorescence, and incomplete glycan release can bias quantitation. For such samples, clean-up procedures (protein precipitation, SPE cleanup) remain essential for maintaining the labelling yield and reproducibility.

#### 5.7.3 Performance and limitations

Instant labeling strategies substantially reduce the sample-preparation time by enabling rapid, reduction-free derivatization with a strong fluorescence and MS response, supporting automation and high-throughput workflows. In practice, their main limitations are operational: labeling is most efficient when performed immediately after enzymatic release because it depends on a freshly opened reducing end, and performance can be sensitive to complex matrices where competing amines, salts, and proteins increase the background signal or reduce yield, necessitating additional cleanup. Finally, widespread adoption is constrained by the proprietary/costly nature of commercial reagents and by the fact that most “instant” chemistries have been optimized and validated primarily for *N*-glycans, with more limited data for *O*-glycans and highly acidic species. Taken together, instant labeling strategies illustrate how derivatization chemistry can shift CE/CE-MS workflows from bespoke methods toward faster, more standardized sample-to-answer formats. However, labeling is only one determinant of analytical performance: the practical robustness and sensitivity of CE-MS ultimately depend on how the labeled (or unlabeled) glycans are separated, ionized, and detected. The next section therefore focuses on CE-MS implementation, covering capillary coatings, background electrolytes, injection strategies, and MS interface designs that govern spray stability, reproducibility, and structural readout.

## 6. CE-MS for glycan analysis

CE-MS has emerged as a powerful platform for glycan characterization, combining exceptional separation efficiency with structural specificity. The technique's ability to resolve closely related isomers and quantify subtle differences in glycan linkages or branching makes it a valuable complement to chromatographic and MALDI-based approaches, offering a valuable perspective on glycan macro- and microheterogeneity.^182^

In CE-MS, analytical performance is governed by three interdependent factors: the environment for electrophoretic separation, the ionization interface, and the chemical nature of the analyte, including any derivatization introduced during sample preparation. Understanding how these factors interact is essential for designing robust, high-sensitivity workflows for glycomics analysis of complex clinical samples.

### 6.1 Principles of CE separation in glycomics

In CE, analytes migrate through a narrow capillary (*e.g.* 30 up to 150 μm internal diameter) under an applied electrical field (∼10–30 kV), separating primarily based on their charge-to-size ratio and interaction with the buffer system. For glycan analysis, this translates into the resolution of species that differ in charge state (*e.g.* sialylation, sulfation or phosphorylation) as well as hydrodynamic volume (*e.g.* core *vs.* antennae fucosylation or branching). Separation can occur within minutes^5,267^ or can take up to 1–2 h;^73^ it requires minimal sample quantities, typically nanoliter (nL) injections containing picogram (pg) or attomole amounts of glycans,^74^ making CE inherently suited to analyzing low-abundant glycans in low-amount clinical samples such as plasma or tissue biopsy materials.

**Fig. 12:**
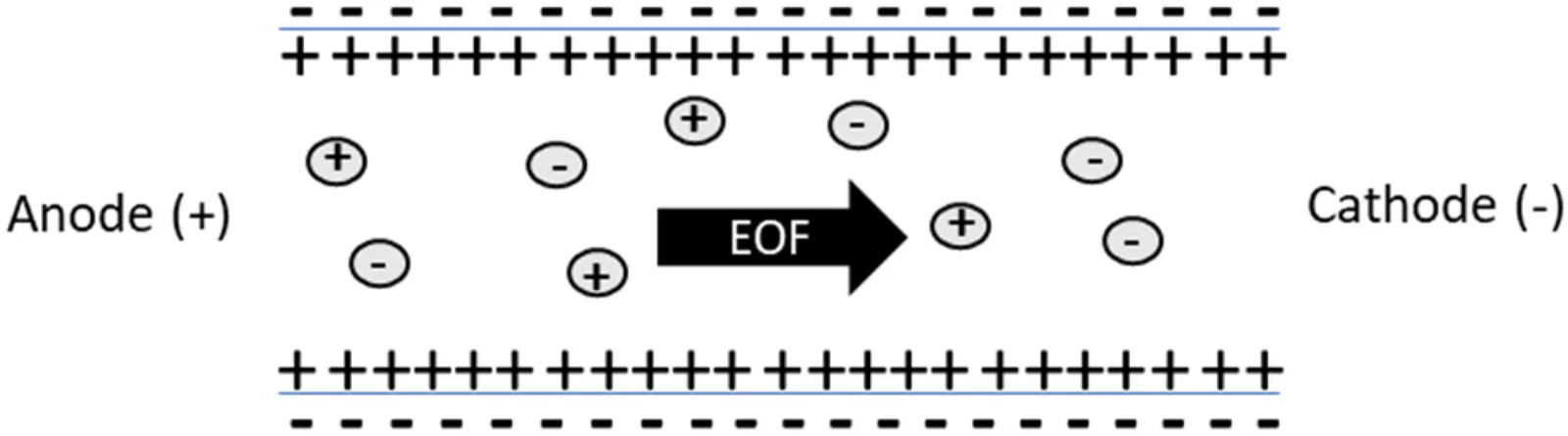
Illustration of electro-osmotic flow (EOF). Under an applied electric field, the movement of solvated counter-ions in the diffuse double layer near the capillary wall generates bulk flow of the buffer solution from the anode (+) toward the cathode (–), carrying analytes toward the detector.

The bulk movement of liquid inside a bare fused-silica capillary is governed by electroosmotic flow (EOF), generated by the ionization of silanol groups on the inner capillary wall (Fig. 12). The magnitude and direction of EOF depends on both the BGE composition, including pH, ionic strength, and buffer species, and the capillary coating, which modifies the surface charge and can suppress analyte adsorption.^5,268^ Neutral or positively charged coatings (*e.g.* polyacrylamide,^269,270^ polyvinyl alcohol,^271^ or linear polyethylenimine^272^) are frequently used to stabilize EOF, reduce wall interactions, and improve migration reproducibility.^270^

Because native glycans are largely neutral, effective CE separation often relies on derivatization, which imparts charge *via* fluorescent or ionic labels (*e.g.* APTS,^5^ ANTS^273^) or through ionizable groups introduced during chemical modification (*e.g.* GirP).^74^ While capillary gel electrophoresis (CGE) has revolutionized protein analysis by providing size-based separations in capillary format,^274,275^ its application in glycomics remains limited due to its incompatibility with MS. The small size and structural similarity of glycans make charge-based separations far more effective. Consequently, most glycan-focused CE workflows rely on capillary zone electrophoresis (CZE),^276^ where analytes migrate freely in solution according to their charge-to-size ratio, or on micellar electrokinetic chromatography (MEKC),^277^ where surfactant micelles act as a pseudo-stationary phase and enable separation through differential hydrophobic partitioning between the micellar core and aqueous buffer; in both cases, separation is driven primarily by charge, polarity, or hydrophobicity introduced through derivatization rather than molecular size alone.^5^ Together, these developments highlight both the analytical power of CE-MS and the remaining technical challenges, which are discussed in the following section.

#### 6.1.1 Injection strategies and quantitative performance

Sample injection is a critical determinant of quantitative accuracy in CE and directly influences detection sensitivity. Two injection modes are commonly used. Hydrodynamic injection introduces a well-defined sample volume through pressure or siphoning and offers excellent reproducibility, but the inherently small injection volumes limit the total analyte load (*e.g.* 2% of capillary volume). By contrast, electrokinetic injection enables much larger effective sample volumes to enter the capillary, improving sensitivity but introducing bias toward highly charged species. This bias can distort the relative glycan profile in the sample, particularly in complex mixtures rich in acidic, sialylated, or derivatized glycans. Optimizing the injection time, voltage, and sample matrix composition is therefore essential when quantitative accuracy is required, especially for low-abundance glycans or clinical samples where matrix effects may be significant.

### 6.2 Coupling CE with MS

**Fig. 13:**
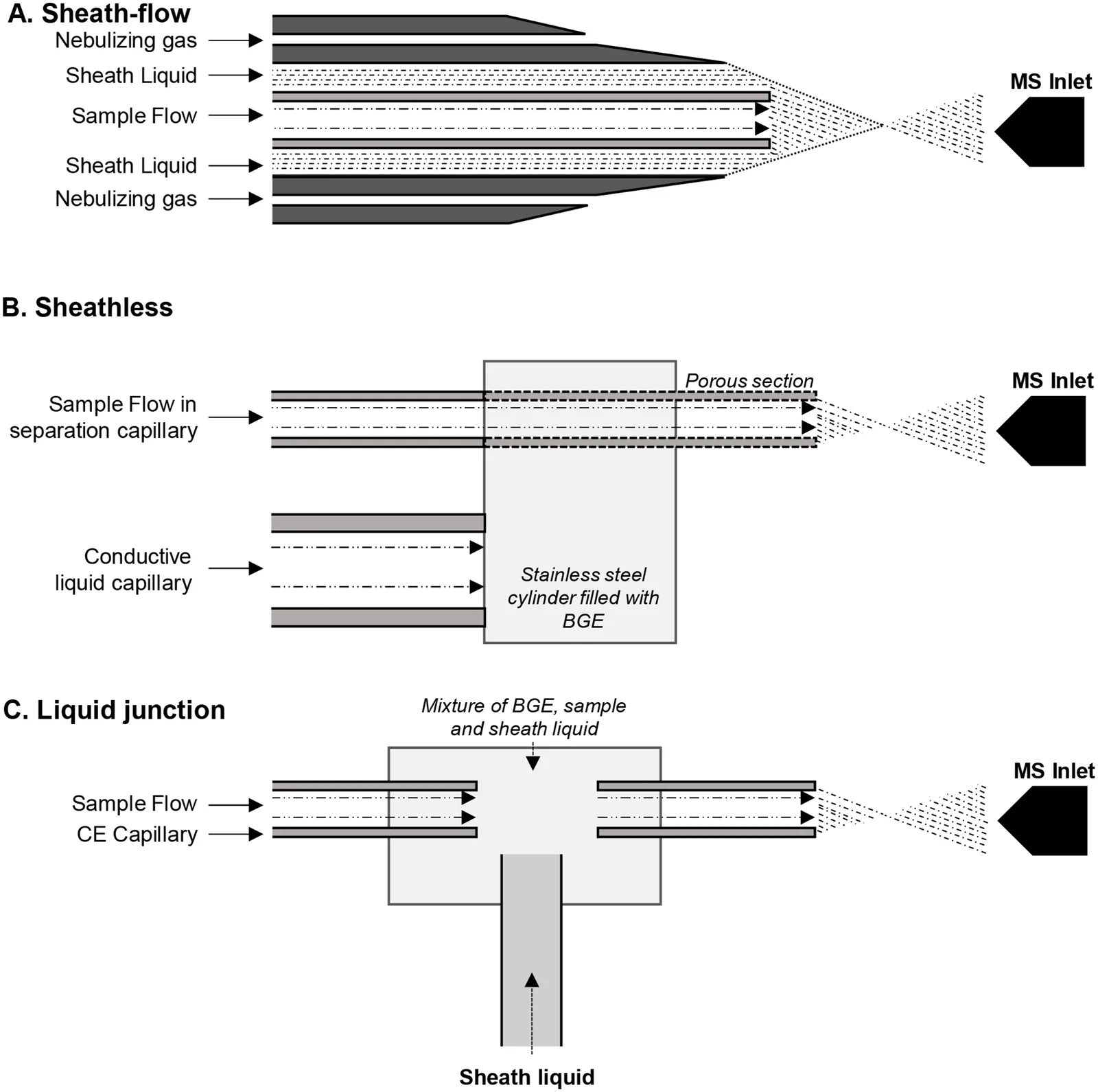
Schematics of the common interfaces for coupling capillary electrophoresis (CE) with mass spectrometry (MS). (a) Sheathflow: the most robust design where a makeup sheath liquid provides electrical connectivity and flow stability for ESI at the cost of some sensitivity lost due to dilution and higher flow rate. (b) Sheathless: offers the highest sensitivity by using the CE separation buffer itself for ESI, with voltage applied directly through a conductive capillary emitter [the figure does not show a separate capillary as it is in the CESI system], but requires more specialized fabrication. (c) Liquid junction: a versatile compromise where a stable electrical connection is maintained through a reservoir of background electrolyte (BGE), balancing ease of use with reasonable performance at the expense of minor band broadening.

Coupling CE to MS (CE-MS) enables the simultaneous achievement of high-resolution electrophoretic separation and molecular-level identification. This integration is particularly advantageous in glycomics, where structural isomers and minor glycoforms differ only subtly in mass or linkage but can produce distinct biological effects. Because CE operates with aqueous, low-flow systems that are inherently compatible with pure electrospray ionization (ESI) (where compound competition for charge is minimal), CE-MS offers a natural route to sensitive, online detection. However, achieving robust coupling requires careful optimization of the interface, often dealing with nL min^−1^ ultra-flow regimes, which governs electrical continuity, solvent compatibility, and ionization stability. Three major interface architectures are used in CE-MS—sheath-flow, sheathless and liquid-junction systems (Fig. 13)—and are further discussed in Sections 6.2.2 and 6.2.3.

#### 6.2.1 Buffer and label requirements for efficient CE-MS coupling

The background electrolyte (BGE) composition and the polarity of glycan derivatization critically influence coupling efficiency in CE-MS. Volatile buffers such as ammonium formate, acetate, or bicarbonate are preferred because they support stable electrospray ionization, whereas non-volatile salts (*e.g.*, borate, phosphate) can promote salt deposition at the emitter, leading to signal suppression and unstable spray, particularly during longer sequences.

The charge of the glycan label further determines the optimal ionization mode. Positively charged (Girard reagents) or cationizable labels (*e.g.*, ProcA and InstantPC) are commonly paired with normal-polarity and positive-ionization mode CE-MS, often yielding strong ionization efficiency and rich glycosidic and cross-ring fragmentation. In contrast, highly anionic labels (*e.g.*, APTS, ANTS) and native acidic glycans are naturally compatible with reversed-polarity CE separation and negative-ionization MS, which can help to preserve sialylation and provide complementary structural information. Optimizing the match between BGE composition, label polarity, and ESI polarity is therefore essential for stable spray formation, high sensitivity, and a reproducible quantitative response.

#### 6.2.2 Sheath-flow interfaces

In sheath-flow configurations, a coaxial solvent stream, typically a mixture of methanol or acetonitrile with volatile electrolytes such as ammonium acetate or formate, surrounds the capillary outlet.^5,278^ This sheath liquid completes the electrical circuit and facilitates electrospray formation. Sheath-flow interfaces are robust, easy to maintain, and compatible with a wide range of BGEs and capillary coatings, making them the most widely implemented design in commercial CE-MS systems. Their main limitation is sample dilution at the outlet, which reduces sensitivity, particularly for low-abundance glycans or small sample volumes.^279^

#### 6.2.3 Sheathless interfaces

Sheathless designs eliminate the external solvent flow and use either a porous-tip emitter or a conductive coating at the capillary terminus to maintain electrical contact. These interfaces operate at nanolitre-per-minute (nL min^−1^) flow rates, improving the ionization efficiency by operating at the regime of pure electrospray with minimal ion suppression and reducing dilution effects. Sheathless CE-MS achieves sensitivity increases of up to one order of magnitude compared with sheath-flow systems, making it particularly valuable for label-free glycan profiling and site-specific glycopeptide analysis, where sample amounts are limited and molecular complexity is greater compared to released glycan analysis. However, sheathless emitters are more delicate and require stringent control of buffer volatility, pH, and cleanliness to prevent clogging or emitter failure.

#### 6.2.4 Liquid-junction interfaces

Liquid-junction interfaces offer an intermediate approach between sheath-flow and sheathless designs. Here, the CE capillary terminates in a small reservoir filled with BGE, which provides a stable electrical connection to the electrospray emitter positioned downstream.^278,279^ This configuration avoids direct coaxial dilution, maintaining higher sensitivity than conventional sheath-flow designs,^279^ while still accommodating a wide range of BGEs and separation conditions. Because electrophoretic and hydrodynamic flows converge in the junction reservoir, a small degree of separation band broadening can occur, and the system detection performance is more sensitive to dead volume than fully integrated sheathless emitters. Nevertheless, liquid-junction interfaces are valued for their robustness, ease of alignment, and broad method compatibility, particularly in environments where separation conditions and MS requirements must be balanced pragmatically.

### 6.3 Performance and recent advances

As CE-MS has matured, its analytical performance has been shaped not only by the intrinsic efficiency of CE but also by continual improvements in capillary coatings, buffer systems, injection strategies, interface engineering, and mass spectrometric detection.^280,281^ Together, these developments have expanded the applicability, robustness, and sensitivity of CE-MS, enabling high-confidence glycan analysis from smaller and more complex samples than previously possible.^74,282^ The following subsections highlight key technical advancements that have driven this progress and outline current performance capabilities within modern CE-MS workflows.

#### 6.3.1 Separation efficiency, sensitivity, and reproducibility

The analytical performance of CE-MS in glycomics is defined by its exceptional separation efficiency, high sensitivity, and capability of resolving structural isomers that often remain indistinguishable by common chromatographic techniques. CE offers theoretical plate numbers exceeding 10^6^, enabling the baseline separation of glycans differing by a single monosaccharide linkage or sialic acid position.^283^ Relative quantitation can be achieved with comparable precision to that of LC-MS,^282,284^ and migration time reproducibility can be further improved to 1–3% RSD when internal standards or migration markers are employed, enabling predictable electrophoretic mobility (*μ*_eff_)^285^ and identification confidence. Detection limits in the low attomole-to-femtomole range have been reported for both fluorescently labelled and native glycans, depending on label type, buffer composition, and interface design.^74^

#### 6.3.2 Advances in capillary coatings and BGE optimization

Recent innovations in capillary coatings and BGE design have substantially enhanced the robustness and reproducibility of CE-MS workflows for glycan analysis. Bare fused-silica capillaries contain ionizable silanol groups that generate strong EOF and readily adsorb charged or hydrophobic analytes, leading to peak distortion, migration-time drift, and capillary instability.^5^ To mitigate these effects, a broad range of dynamic and covalently bonded coatings have been developed. Linear polyacrylamide (LPA) and polyvinyl alcohol (PVA) coatings effectively suppress wall interactions and provide highly stable EOF, often over hundreds of injections, while maintaining compatibility with MS-friendly (*e.g.*, acetic acid, ammonium acetate, and ammonium formate^5,74^) volatile buffers.^270,286^ Other coatings, such as polyethylenimine (PEI), enable the direction and magnitude of EOF to be tuned for particular glycan classes or negative-mode MS operation.^287^ Emerging zwitterionic and PEG-based coatings have further contributed to reducing adsorption and improving long-term reproducibility, particularly in complex matrices such as serum or plasma.^288^

Parallel advances in BGE formulation have played an equally important role in improving CE-MS performance. Modern CE-MS methods rely almost exclusively on volatile electrolytes, most commonly ammonium formate, ammonium acetate, or ammonium bicarbonate, because they support stable electrospray formation and minimize emitter fouling. The careful use of organic modifiers such as methanol or acetonitrile lowers surface tension and enhances desolvation, leading to improved ionization efficiency and sharper peak shapes, while small amounts of formic acid provide additional control over the charge state and spray stability.^5,74,289^ Together, these improvements in coating chemistry and electrolyte formulation create an electrophoretic environment that is highly reproducible and fully compatible with efficient ESI, enabling the high separation efficiency of CE to be translated effectively into MS-based glycan analysis.

#### 6.3.3 Interface and ionization innovations

Electrospray interface design remains one of the most decisive factors influencing CE-MS sensitivity, robustness, and practical usability. A major source of performance improvement in CE-MS has come from the adoption of nanoflow electrospray ionization (nanoESI). Compared with conventional ESI, nanoESI operates at substantially lower flow rates (typically tens to hundreds of nL min^−1^),^290^ producing smaller initial droplets with more efficient desolvation, without the requirement for an additional spraying support such as use of high-speed nebulizing gas.^291,292^ This reduces sample dilution at the source and generally enhances ionization efficiency, leading to improved sensitivity for low-abundance glycans. The gentler spray conditions can also mitigate in-source fragmentation of labile structures, such as sialylated, sulfated, or phosphorylated glycans, although preservation of these residues ultimately depends on the ionization mode, BGE composition, and source parameters rather than on a low rate alone. NanoESI, however, due to a low eluent speed, normally requires more delicate emitter geometries and precise alignment with the MS inlet, making some commercial implementations technically demanding.

To address these challenges, nanoflow sheath-liquid interfaces have been developed as a compromise between the robustness of traditional sheath-flow systems and the sensitivity of sheathless designs. By supplying only a minimal sheath-liquid flow, these interfaces maintain electrical continuity while significantly reducing sample dilution. Recent advances include the nanoCEasy interface developed by Neusüß and co-workers,^293^ which uses a 3D-printed assembly to hold the separation capillary, emitter, and electrode without screws or precision fittings. This simplified design reduces the mechanical complexity while maintaining stable low-flow ESI, offering nanoflow performance without the fragility of porous-tip sheathless emitters. Such innovations have made high-sensitivity CE-MS more reproducible and extends glycomics analysis to applications requiring attomole-level detection.

**Fig. 14:**
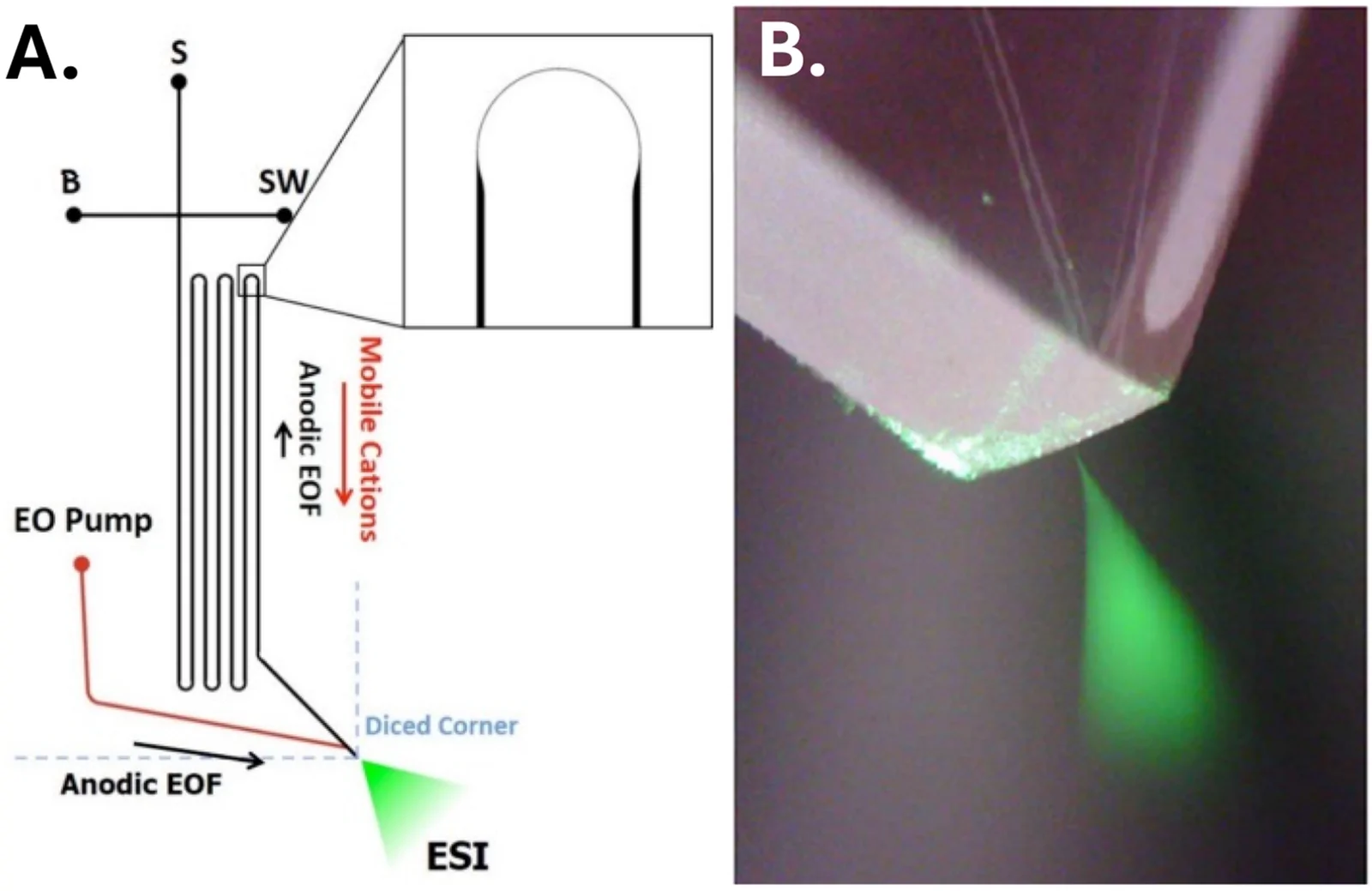
Schematic of the integrated microfluidic CE-ESI-MS interface (ZipChip). (A) The device comprises a monolithic microfluidic device incorporating the sample (S), background electrolyte (B), sample waste (SW), and electroosmotic pump reservoirs (EOs), which are connected to a 23 cm separation channel and an integrated nanoelectrospray emitter. The enlarged inset shows the asymmetric turn tapering near the emitter region. Red channels indicate the APS coating, whereas black channels indicate the APS-PEG450 coating. (B) Photograph of the electrospray plume generated from the corner of the device at the integrated ESI emitter, visualized using a green laser. Reprinted with permission from Redman, Batz, Mellors, and Ramsey, *Anal. Chem.*, 2015, **87**(4), 2264–2272, https://doi.org/10.1021/ac503964j (Fig. 1 (A) & Fig. S1 (B)). Copyright 2015 American Chemical Society.^62^

Microfluidic CE-MS platforms have significantly simplified and accelerated the implementation of CE-MS for biomolecular analysis^294^. Devices such as the ZipChip (908 Devices;^295,296^Fig. 14) and the Blaze system (originally Intabio,^297^ now developed and commercialized by SCIEX) integrate the separation channel, nanoESI emitter, grounding structures, and fluidic reservoirs into a single disposable microchip. This design eliminates manual capillary alignment, minimizes dead volume, and provides an exceptionally stable nanoESI performance. As a result, these systems routinely achieve rapid separations, typically 2–10 min per sample, with high reproducibility and minimal operator intervention.

While their absolute sensitivity is lower than that of state-of-the-art sheathless porous-tip emitters, microfluidic CE-MS platforms offer unmatched ease of use, robustness, and throughput. ZipChip has become widely adopted for intact protein, metabolomic, and glycan profiling due to its plug-and-play cartridge format.^99,298^ Together, these platforms demonstrate how microfluidic CE-MS can bridge discovery, screening, and quality control (QC) oriented applications by providing fast, reproducible, and user-friendly separations without the need for specialized CE expertise.

**Fig. 15:**
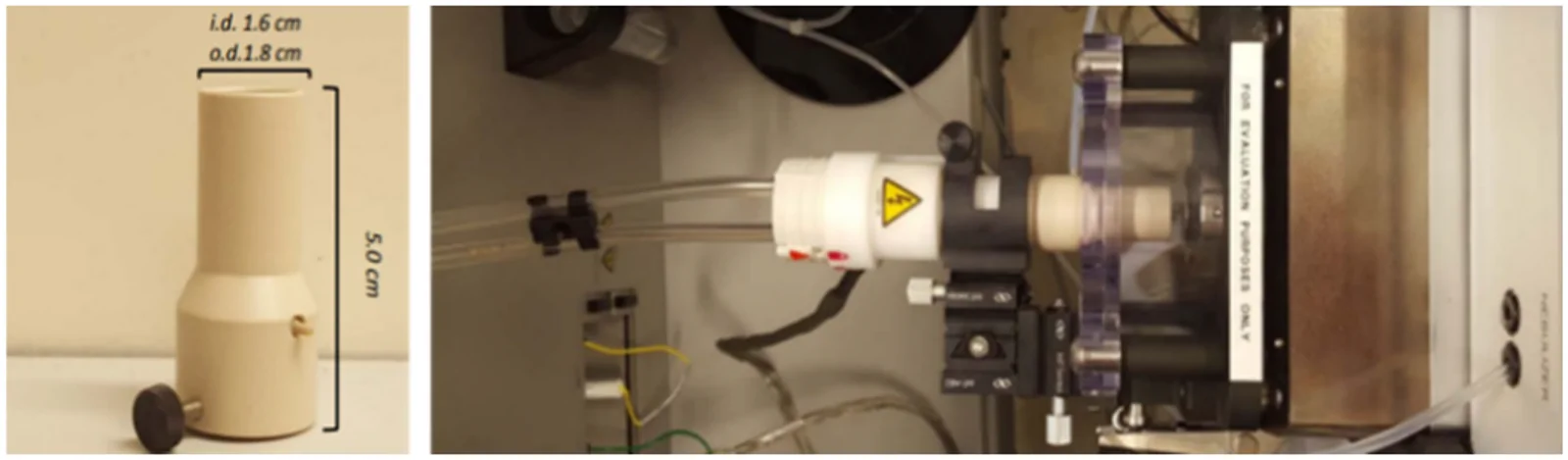
Schematic and implementation of the in-house fabricated dopant enriched nitrogen (DEN) gas connection interface for sheathless CE-ESI-MS. The ionization chamber is continuously purged with a flow of nitrogen gas enriched with a suitable organic solvent dopant (*e.g.*, isopropanol or acetonitrile). Reproduced from Kammeijer, G. S. M., *et al.*, with permission from the American Chemical Society.^282^

Further improvements in ionization have been enabled by dopant enriched nitrogen (DEN) sheath gas (Fig. 15), which increases the local electric field strength at the emitter tip and promotes finer droplet formation and increases charge states.^282^ In glycomics applications, DEN-gas has produced approximately 3.3-fold increases in signal-to-noise ratios while maintaining relative standard deviations of glycan signal intensity below 4%, significantly boosting the sensitivity for low-abundance glycans and enhancing the detectability of low-abundance glycans and glycopeptides.^74^ Importantly, these gains are achieved without altering the electrophoretic separation itself.^75^

Collectively, advances in emitter design, nanoESI operation, microfluidic integration, and sheath-gas engineering have transformed CE-MS from a specialized research tool into a far more versatile and sensitive platform for high-resolution glycan analysis.

#### 6.3.4 Advances in mass spectrometry and automation

Recent developments in MS have significantly strengthened the analytical capabilities of CE-MS. Modern high-resolution mass analyzers, including time-of-flight (TOF), Orbitrap, and Fourier-transform ion cyclotron resonance (FT-ICR), provide sub-ppm mass accuracy, a large dynamic range (10^6^–10^9^), and a high scanning speed (up to 270 spectra in Astral Zoom analyzer) and resolving power, allowing confident discrimination of subtle glycan variants and co-migrating species. When coupled to CE's sharp, peak profiles, these instruments now enable structural annotation directly from individual electrophoretic peaks, often without the need for additional fractionation.

Fragmentation technologies have also advanced. While CID/HCD remains widely used for glycan glycosidic cleavage, electron-based fragmentation methods such as ETD,^258^ EAD^259^ and ECD^299^ provide complementary information by generating cross-ring fragments that reveal linkage positions and branching patterns.^300^ This combination is particularly important for resolving isomers with structural differences that lie in subtle linkage rearrangements.

Automation and throughput have progressed in parallel. Devices such as the ZipChip system integrate pre-filled separation channels, fixed nanoESI emitters, and automated electrolyte conditioning and emitter MS interface alignment routines, greatly reducing the hands-on time and improving run-to-run reproducibility. These chip-based formats also incorporate on-chip desalting or buffer-exchange functionality, enabling the direct injection of moderately complex samples. As a result, analysis times of 2–10 min per sample are achievable, making such platforms attractive for biopharmaceutical workflows and high-throughput glycomics. In contrast, conventional CE-MS systems (sheath-flow or sheathless capillaries) do not yet offer comparable automation, and separation times remain highly method-dependent.

Multi-segment and serial injection strategies enable several samples to be analyzed within a single electrophoretic run,^286,301^ substantially increasing throughput while maintaining separation integrity. Pre-filled interface cartridges and automated conditioning modules streamline system preparation and reduce operator-dependent variability, facilitating more robust method transfer and routine implementation. These developments have brought typical analysis times to under 15 min per sample, making CE-MS increasingly suitable for biopharmaceutical quality control and medium-throughput glycomics.

Improvements in data processing are beginning to contribute to performance gains in glycomics, although this area is less mature than in proteomics.^302,303^ Tools for migration-time alignment, peak picking, and spectral deconvolution, often adapted from adjacent MS fields, together with new developments such as PASTAQ,^304^ OpenMS^305^ and mzMine,^306^ can improve feature detection, resolve partially overlapping signals (*e.g.*, co-migrating glycoforms and isotope/adduct clusters) and reduce peak quantification errors. These gains are particularly evident when advanced fitting approaches, such as wavelet or Gaussian-based methods, are used instead of standard XIC-based one-dimensional peak integration.^307,308^ In parallel, emerging annotation workflows that combine migration time (or calibrated mobility), accurate mass, and MS/MS fragment annotation can improve identification confidence and consistency, particularly when supported by curated libraries and standardized QC metrics.^309,310^ GlycoGenius software represents a notable example of an end-to-end glycomics data analysis tool, supporting workflows from automated glycan composition library generation to MS/MS-based annotation within a single platform.^311^

Despite these advances, challenges remain. Quantitative accuracy continues to be influenced by injection variability, requiring careful optimization of hydrodynamic or electrokinetic injection, depending on sample complexity and glycan charge distribution. CE capillaries and nanoESI emitters remain susceptible to contamination or fouling, necessitating rigorous maintenance to ensure long-term reproducibility. Matrix effects, particularly in plasma, serum, or tissue extracts, can suppress ionization or alter electrophoretic behavior, underscoring the need for effective sample cleanup and enrichment strategies. Method transfer between laboratories also remains non-trivial due to differences in capillary coatings, emitter geometry, and instrument tuning. However, migration time alignment algorithms, including two-dimensional warping approaches such as the Warp2D^312^ algorithm originally developed for LC-MS data, together with internal mobility markers and software-based normalization strategies such as total area normalization (%area), internal standard (IS)-based normalization, and total ion current (TIC) normalization,^17,286,313^ can efficiently compensate for minor migration time fluctuations^285^ and improve cross-run comparability.

Recent progress in capillary-coating chemistry, integrated chip-based interfaces, spray-stabilizing dopant/enrichment gases, and automated migration-time correction is steadily reducing these bottlenecks. Collectively, these innovations are positioning CE-MS as a highly sensitive, structurally informative, and increasingly robust platform for both targeted and discovery-driven glycomics.

### 6.4 Applications

CE-MS has become a valuable tool for the structural analysis of released glycans in biomedical research and biopharmaceutical development.^74,75,314^ Its unrivalled separation efficiency enables the resolution of isomeric and closely related glycan structures that frequently co-elute in RP, HILIC or PGC chromatography. This capability is particularly important for differentiating α2-3 *versus* α2-6 sialylation, core- *versus* outer-arm fucosylation, or variations in LacNAc extension that may influence immune recognition or therapeutic activity.

In quantitative glycomics, CE-LIF remains one of the most sensitive and reproducible profiling techniques. When paired with CE-MS, fluorescence-based absolute quantitation can be combined with structural confirmation, enabling robust comparative studies across large sample cohorts.^74,315^ CE is especially well suited to clinical glycomics due to its low sample consumption and ability to resolve diagnostically relevant isomeric features in tissue and body fluid such as plasma, serum, saliva or cerebrospinal fluid (CSF).^316,317^

In biopharmaceutical analysis, CE-LIF is widely used for batch comparison of *N*-glycans released from monoclonal antibodies and Fc-fusion proteins, while CE-MS adds structural depth and improves the detection of low-abundance glycoforms.^318^ The rapid enzymatic release kits currently available and instant labelling chemistries enable complete CE-based glycan analysis on timescales compatible with process development and quality control.

### 6.5 Comparative outlook: CE-MS *vs.* other glycomics platforms

In comparison with HILIC-MS, CE-MS offers superior separation efficiency for ionic and more polar glycans, particularly those differing in sialylation, charge, or subtle branching patterns. HILIC excels in robustness and ease of method transfer but often struggles to resolve positional isomers. PGC provides excellent isomer resolution but at the expense of longer run times, retention-time instability, and potential sensitivity loss for very hydrophilic structures. CE, by contrast, requires minimal sample preparation when MS-compatible buffers are used. These characteristics position CE-MS as a valuable orthogonal technique for complementing LC-based workflows.

Compared with MALDI-TOF-MS, CE-MS offers greater structural specificity. Conventional MALDI-TOF/TOF platforms provide unparalleled speed and throughput but are limited in sensitivity and fragmentation detail, relying on low-energy CID, which often yields insufficient structural information for linkage assignment.^58^ However, modern atmospheric pressure (AP)-MALDI sources coupled to Orbitrap instruments such as the Masstech AP-MALDI source enable access to higher-energy fragmentation methods, including HCD and ETD, substantially improving structural specificity.^319,320^ Nevertheless, challenges in distinguishing isomers without derivatization remain across most MALDI platforms. CE-MS, particularly when combined with electron-based fragmentation (EAD/ETD/ECD), enables detailed linkage and branching assignment directly from separated peaks. However, MALDI remains advantageous for large sample sets, tissue imaging, and applications where speed outweighs the need for full structural resolution.

Despite its clear strengths, CE-MS still faces challenges. Capillary surface instability, emitter fragility, and susceptibility to matrix effects can impact reproducibility, particularly in multi-lab settings. Sensitivity can be higher than that in LC-MS when sheathless interfaces are used but may be lower with sheath-flow systems^279^ or when using hydrodynamic injections.^98^ In addition, the sample loading capacity remains limited, as typically only 1–10% of the available sample can be injected, restricting sensitivity for low-abundance analytes and low sample volumes such as single cells or laser microdissected cell sections. Moreover, method standardization and inter-instrument reproducibility remain areas where CE-MS requires further development before achieving ubiquitous adoption in regulated environments.

Nevertheless, continuous improvements in capillary coatings, nanoflow electrospray designs, microfluidic CE-MS cartridges, and migration-time normalization algorithms are rapidly strengthening its robustness and accessibility. As the technology matures, CE-MS is increasingly positioned not only as an orthogonal supplement to LC-MS and MALDI-MS, but as a standalone high-resolution analytical platform capable of providing unique structural insights in both research and applied glycomics.

## 7. Discussion and future directions

The maturation of CE-MS has been driven by parallel advances in derivatization chemistry, capillary technology, MS interfacing and MS selection and fragmentation technologies. These components are deeply interconnected: improvements in one domain often reveal limitations in another. When optimally aligned, CE-MS provides structural resolution that is difficult to achieve with any other platform. Yet this same interdependence contributes to method complexity and is a major source of variability between laboratories.

A central challenge for the field is the need for greater standardization and robustness and assessment of interlaboratory performance. In contrast to LC-MS, which benefits from decades of harmonization, CE-MS workflows still vary widely in capillary coatings, BGE composition, injection strategies, and interface configurations. The development of universal coatings, validated MS-compatible BGEs, and migration-time calibration standards would significantly improve reproducibility and enable the broader adoption of CE-MS analysis in laboratories with low levels of CE expertise.

Workflow automation represents another important frontier. Microfluidic CE-MS platforms, including ZipChip and IntaBio cIEF-MS,^297^ demonstrate how integrated, cartridge-based architectures can stabilize the nanospray, reduce manual intervention, and shorten analysis times. As these systems advance, they are likely to play a key role in translating CE-MS from specialized research groups to routine biomedical and biopharmaceutical environments.

A recurring theme throughout this review is the intimate interplay between derivatization chemistry, separation behavior, and MS fragmentation. Labels that enhance fluorescence or ionization can also reshape electrophoretic mobility, influence migration-time reproducibility, alter adduct patterns, and affect the stability of labile residues. There is a clear need for next-generation derivatization strategies that combine the speed of instant labels with the quantitative reliability of reductive amination, while also supporting informative MS/MS fragmentation. Ideally, such chemistries would extend beyond *N*-glycans to encompass other glycan classes, including *O*-glycans, released glycosphingolipids, and glycosaminoglycans, and provide modular or orthogonally reactive handles for multiplexing (*e.g.* TMT), enrichment, or enhanced structural elucidation.

Looking forward, progress in several areas will be essential for strengthening the robustness and accessibility of CE-based glycomics. Advancements and the combined use of long-lasting, MS-compatible capillary coatings; improved sheathless and conductive-polymer emitters; dopant enrichment gases; and chip-integrated CE-MS cartridges will all contribute to improved stability and ease of implementation. Complementary developments in derivatization chemistry, interface design, and data processing will help align CE-MS with the operational expectations of high-throughput scientific and industrial laboratories.

Overall, these developments are converging on the same goal: CE-MS workflows that are sensitive yet routine. In the near term, the most impactful gains will likely come from standardized, MS-compatible method “recipes” (coatings, BGEs and migration-time calibration), coupled with more stable emitter designs and cartridge-based implementations that reduce operator dependence. In parallel, derivatization strategies that preserve the quantitative performance while improving MS/MS interpretability, and that can be generalized beyond *N*-glycans, will further expand the scope of CE-based glycomics.

## 8. Conclusions

CE-based glycan analysis, particularly when coupled to MS, combines high-efficiency separations with structural specificity, enabling the resolution of glycan isomerism and microheterogeneity from limited sample amounts. Recent advances in reducing-end derivatization, MS-compatible electrolytes and interface engineering have improved the sensitivity and reproducibility of glycan profiling and expanded the practical operating space for CE-MS. Although still more specialized than chromatographic platforms, CE-MS has evolved into a mature, highly sensitive and structurally powerful technique, the unique strengths of which complement those of LC-MS and MALDI-MS. CE-MS is poised to assume an increasing central role in comprehensive glycan characterization across biomedical research, clinical studies, and biopharmaceutical development.

## Author contributions

Karthika Korumadathil Shaji: conceptualization, investigation, resources, supervision, visualization, writing – original draft, writing – review & editing. Peter L. Horvatovich: conceptualization, funding acquisition, resources, supervision, writing – original draft, writing – review & editing, Guinevere S. M. Lageveen-Kammeijer: conceptualization, funding acquisition, investigation, resources, supervision, visualization, writing – original draft, writing – review & editing.

## Conflicts of interest

There are no conflicts to declare.

## Data Availability

No primary research data were generated or analyzed in this study. All data supporting this review are from previously published sources cited in the article.
